# Feasibility and safety of remote robotic hepatectomy: a prospective single-arm study with MP1000 system in China

**DOI:** 10.1016/j.eclinm.2025.103579

**Published:** 2025-10-22

**Authors:** Wei Liao, Hai-Tao Zhu, Yu-Ting Guo, Gen Cheng, Nan Qiao, Bi-Xiang Zhang, Xiao-Ping Chen, Peng Zhu

**Affiliations:** a Hepatic Surgery Center, Tongji Hospital, Tongji Medical College, Huazhong University of Science and Technology, Wuhan, Hubei, China; b Department of Hepatobiliary Surgery, The Affiliated Hospital of Guizhou Medical University, Guiyang, Guizhou, China; c Guizhou Institute of Precision Medicine Biobank, Guiyang, Guizhou, China; d Shenzhen Edge Medical Co., Ltd., Shenzhen, Guangdong, China

**Keywords:** Telesurgery, Robotic, Hepatectomy, Remote

## Abstract

**Background:**

Telesurgery has demonstrated many advantages in certain scenarios. However, given the high risks of liver resection, there have been no prospective studies to evaluate the safety and effectiveness of robotic remote liver resection. This study aimed to evaluate the safety and effectiveness of the MP1000 remote surgical system in robot-assisted remote liver resection using the first prospective single-arm cohort study.

**Methods:**

This study was approved by the Clinical Trial Ethics Committee of Huazhong University of Science and Technology ([2024] (324)) and registered at Chinese Clinical Trial Registry (ChiCTR2500097679). Six patients were continuously recruited from March 1 to April 18, 2025. After strict preoperative evaluation, robotic liver resection was performed using the MP1000 remote surgical system. The primary outcomes were the surgical success rate and the incidence of complications. The postoperative task load of surgeons was assessed using the NASA–TLX scale.

**Findings:**

The network connection of the remote system was smooth. All surgeries were successfully completed without conversion to open surgery or local robot—assisted surgery. Surgeons reported a relatively low workload. All patients recovered smoothly and were discharged. However, the second patient developed bacteremia within 30 days after surgery and was readmitted but recovered after antibiotic treatment.

**Interpretation:**

Robotic remote liver resection is a feasible and effective minimally invasive treatment modality that can provide high-quality medical services to areas with constrained medical resources. Nevertheless, further multi-center, large-scale randomized controlled trials are required to verify its effectiveness and safety.

**Funding:**

This work was supported by the Noncommunicable Chronic Diseases- National Science and Technology Major Project (2023ZD0502001), 10.13039/501100001809National Natural Science Foundation of China (82473040), Hubei Province Key Technology Breakthrough Project (2023BAA016-3), Tongji Hospital Clinical Research Fund (2024TJCR014).

**Research in context**

**Evidence before this study**

Our team's series of studies have demonstrated that localized robotic liver resection is safe, feasible, and characterized by a short learning curve. In patients with very early or early-stage hepatocellular carcinoma (HCC), this approach achieves long-term oncological outcomes comparable to those of open hepatectomy. All patients were enrolled based on guideline-recommended surgical criteria. Robust evidence supports the safety and efficacy of localized robotic liver resection—notably, the MP1000 surgical robotic system has already been successfully applied in remote urological procedures, and preclinical animal studies have confirmed the feasibility of remote hepatectomy.

**Added value of this study**

Through a small-scale exploratory clinical trial, we provide the first clinical evidence that remote hepatectomy is a safe and effective minimally invasive approach for both benign and malignant liver diseases. This breakthrough technology can deliver high-quality surgical care to medically underserved regions and facilitate large-scale expertise sharing and surgical skill training among specialists.

**Implications of all the available evidence**

Remote liver resection has been demonstrated as a feasible and effective minimally invasive approach for the treatment of both benign and malignant hepatic conditions. Nevertheless, further validation via multicenter, large-scale randomized controlled trials (RCTs) is imperative to confirm these initial results, develop standardized operational protocols, and facilitate the integration of remote liver resection into mainstream clinical practice.

## Introduction

Robot-assisted surgery has gained widespread acceptances for its precise and minimally invasive approach, naked eye 3D technology, high dexterity and short learning curve.^1^ The first clinical telesurgery, known as the famous Lindbergh operation, was performed in 2001 across the Atlantic ocean with ZEUS robotic system and the transatlantic optical fiber network.^2^ Although high operational costs, unsatisfied network bandwidth and delay, and unclear reliability were all prominent disadvantages of initial stage of telesurgery, it was still a milestone in surgery history. However, the development of telesurgery was at a standstill for these disadvantages, lacking industry support and commercial investment.^3^ With the rapid development of surgical robotics and network communication technology in the recent two decades, the exploration of telesurgery has come true and given people hope for the advantages of overcoming geographical barriers to provide high-quality medical service in selective surgical technology-deficient regions, optimizing medical resource distribution.^4^, ^5^, ^6^

Domestic robotic telesurgery has also gone through a rather difficult process. However, excessively high latency rates and significant packet loss rates still limit the popularization and application of this technology.^7^^,^^8^ However, high speed with low delay 5G communication technology, cooperating with novel advanced surgical robotic platforms and experienced surgeons, reignited the hope for the application of telesurgery again. Liu tried domestic robotic system with 5G communication technology to perform world's first animal remote liver resection and gained a satisfied latency <150 ms in a distance of 50 km and no any adverse event of software or hardware occurred in the experiment.^9^ Afterwards, there were occasional reports of a small number of individual remote surgeries, mainly in the field of urological surgery.^10^, ^11^, ^12^, ^13^ In 2022, on the base of robot-assisted liver resection was safe, feasible and achieving comparable long-term oncological outcomes to open hepatectomy for patients with very early/early stage hepatocellular carcinoma (HCC), we took the lead in conducting the registration clinical trial of the MP1000 surgical robotic system (Shenzhen Edge Medical Company, Shenzhen, China) for hepatobiliary surgery and successfully obtained the qualification for the indication of hepatobiliary surgery in China.^1^^,^^14^ Based on the MP1000 remote surgical system, we initiated the world's first registration clinical study on remote MP1000 surgical robotic hepatectomy, approved by National Medical Products Administration (NMPA), to evaluate the safety and effectiveness of remote robotic hepatectomy.

In this study, we established a prospective cohort to perform remote robotic liver resection (the surgeon console was in Wuhan, Hubei, China; the patient side cart was in Guiyang, Guizhou, China). After a short-term follow-up, the intraoperative and postoperative data were collected to evaluate the feasibility, safety, and effectiveness of the MP1000 remote system in the process of robotic remote hepatectomy.

## Methods

### Patient selection and surgical team

The prospective cohort was established and maintained since March 1, 2025. All included patients underwent remote liver resection in the Affiliated Hospital of Guizhou Medical University, Guiyang, Guizhou, China. The inclusion criteria were as follows: 1) Aged 18–80 years old, inclusive, with no gender restrictions; 2) Body-Mass Index (BMI) ranging from 18 to 30 kg/m^2^; 3) Possess relevant indications for liver surgery; 4) Physiological conditions are suitable for undergoing laparoscopic surgery; 5) Willing to cooperate and complete the follow-up research as well as related examinations; 6) Voluntarily sign the informed consent form; 7) An indocyanine green retention rate at 15 min (ICG-R15) <15% and Child-Pugh class A. The exclusion criteria were as follows: 1) Individuals afflicted with severe cardiovascular disorders that preclude them from undergoing surgery; 2) Pregnant or lactating women; 3) Persons with a medical history of epilepsy or mental disorders; 4) Severe allergic diathesis, as well as those suspected of or diagnosed with alcohol or drug dependence; 5) Individuals incapable of comprehending the research requisites or failing to fulfill the research follow-up protocol; 6) Those whom the researchers deem ineligible to partake in this trial. The selection of cases is carried out in accordance with the patient's autonomous wishes after the consultation between the patient and the attending doctor. The console surgeon has already overcome the learning curves of robot-assisted liver resection and has extensive experience in robotic and laparoscopic hepatectomy. The assistant surgeon has experienced more than 20 robot-assisted liver resections. In addition, the surgical team also includes a network support team, located in Wuhan and Guiyang respectively, to ensure smooth network connection and testing, and to monitor and maintain the security of the intraoperative network link in real time.

### Ethics

This prospective single-arm study was approved by the Clinical Trial Ethics Committee of Huazhong University of Science and Technology (Approval Number: [2024] (324)). The study protocol was registered at Chinese Clinical Trial Registry (ChiCTR2500097679, Date of Registration: Feb 24, 2025, URL: https://www.chictr.org.cn/showprojEN.html?proj=262926). All eligible patients were enrolled after signing the written informed consent forms.

### Preoperative assessment

Every patient underwent a comprehensive pretreatment evaluation, encompassing laboratory examinations and medical imaging assessments. Specifically, the laboratory examinations consisted of a complete blood count, urinalysis, liver and renal function tests, coagulation assays, and serological tests for hepatitis B and C antibodies. When a tumor was suspected of being malignant, tumor markers such as serum alpha-fetoprotein (AFP), des-gamma-carboxy prothrombin (DCP), carcinoembryonic antigen (CEA), and carbohydrate antigen 19.9 (CA19-9) were further analyzed. Medical imaging evaluations comprised thoracic computed tomography (CT), abdominal ultrasonography, abdominal contrast-enhanced CT, and/or contrast-enhanced magnetic resonance imaging (MRI) of the liver to evaluate liver vasculature and determine tumor resectability. An ICG-R15 test was routinely conducted for every patient to assess their hepatic functional reserve. Three-dimensional reconstructions of the liver were carried out to evaluate major blood vessels and residual liver volume in each case. All cases were deliberated upon at a multidisciplinary team meeting, which included the console and assistant hepatobiliary surgeons, radiologists, and anesthesiologists.

After a consensus was reached during the multidisciplinary meeting, the patients were scheduled for telesurgery.

### Surgical procedures

After the MP1000 remote surgical system is connected, it is necessary to check whether the network bandwidth for data traffic transmission detection is stable and sufficient. The surgical procedures were performed as we previously described.^14^ The patient would be placed in a supine position (for left hemihepatectomy, left lateral lobectomy, resection of segment 5, regional lymphadenectomy, and biliary exploration, etc.). The camera port was positioned on the upper side of the umbilicus. Subsequently, a carbon dioxide pneumoperitoneum was established and the pressure was maintained at 12 mmHg. The MP1000 robotic system was docked on the left side of the patient, and the surgical instruments were installed accordingly. Routine abdominal exploration and intraoperative ultrasound examination were carried out. During the procedure, the Pringle's maneuver clamping was routinely performed. We used an ultrasonic scalpel to perform liver parenchymal transection and ligated or clipped any vessels >2 mm and bile ducts. After hemostasis, the specimen was extracted and one or more drainage tubes were placed on the surface of the raw liver. The surgical team consisted of two surgeons. One served as the console surgeon located in Wuhan, Hubei Province, while the other, the assistant surgeon, was responsible for tasks such as exchanging robotic instruments, delivering surgical supplies, and extracting the specimen in Guiyang, Guizhou Province. A spare console was prepared at the Affiliated Hospital of Guizhou Medical University to address potential situations such as communication interruptions, uncontrolled bleeding, or failure of the main console.

### Outcomes

The primary outcomes of this study were the surgical success rate and the incidence of complications ≥ Clavien-Dindo grade 3. Intraoperative outcomes, such as operative time, surgeon console time, blood loss, R0 resection rate, blood transfusion, instrument defect events, and both subjective and objective evaluations of telesurgery, were considered. Postoperative task load was assessed using the NASA-TLX scale for surgeons, with real-time evaluations of image quality, audio clarity, and instrument functionality for each remote surgery session. Postoperative outcomes, including the overall complication rate, postoperative hospital stay, 30-day readmission rate, reoperation rate, serious adverse event rate, and the textbook outcome in liver surgery (TOLS), were regarded as the secondary outcomes.

### Postoperative care

Full blood cell counts, liver and renal function tests, as well as coagulation tests, would be routinely conducted on postoperative days 1, 3, and 5. Postoperative morbidity and mortality were assessed according to the Clavien-Dindo classification.

### Short-term follow-up

Full blood cell counts, urinalyses, liver and renal function tests, coagulation tests, thoracic CT, and abdominal contrast-enhanced CT would be carried out within one month after surgery.

### Definitions

Postoperative liver failure was defined as a condition in which, on postoperative day 5, the prothrombin time was less than 50% of the normal value and the serum total bilirubin level exceeded 50 μmol/L. Surgical success was defined as the successful completion of a remote liver resection using a complete robotic approach. Round trip time (RTT) was defined as the time interval between the transmission of a data packet from the current address to a specific destination address and the receipt of its return. Operative time was calculated as the time elapsed from the initial incision of the skin to the completion of skin suturing. Surgeon console time referred to the total time the surgeon spent at the console performing the surgical operation. The evaluation of telesurgery encompassed both subjective and objective components. The subjective evaluation involved the assessment of the difficulty degree, which was measured using the subjective mental effort questionnaire. Additionally, the subjective evaluation included the degree of satisfaction with the remote operation, as well as the quality, real-time nature, and stability of the surgical image and sound. In contrast, the objective evaluation comprised metrics such as the mean RTT, mean network jitter, mean video encoding and decoding latency, and packet loss rate. The TOLS was defined as the absence of intraoperative incidents graded ≥ grade 2, postoperative bile leaks graded ≥ grade B, and Clavien-Dindo complications graded ≥ 3 A.^15^ It also required the absence of readmission within 30 days after discharge, in-hospital mortality, and positive surgical margins.

### Statistical analysis

This study was designed as a prospective, single-arm interventional investigation. All clinical data were meticulously recorded in a prospectively structured data sheet. Network latency data was collected every second and presented with line chart. Subjective evaluation scores were presented with scatter diagram with 95% confidence interval, while objective evaluation were presented with radar map. All statistical analyses were conducted using with EXCEL version 16.0.4954.1000 (Microsoft Corp., Redmond, WA, USA) and the Graphpad Prism version 7.0 (Graphpad Software, Boston, MA, USA).

### Role of funding source

The funder had no role in the study design, data collection, data analysis, interpretation or manuscript writing.

## Results

### Baseline characteristics of the patients

The study recruiting period was from March 1, 2025, to April 18, 2025. This study enrolled a total of six patients, meeting the sample size requiring in the clinical trial protocol and comprising three cases with benign diseases and three with malignant neoplasms. All patients underwent preoperative multidisciplinary team (MDT) discussions via teleconferencing systems prior to surgery. Demographic and clinical characteristics, including patient gender, age, and hepatic functional status, were systematically summarized in Table 1.

**Table 1:** Baseline characteristics of patients.

Parameters	Patient 1	Patient 2	Patient 3	Patient 4	Patient 5	Patient 6
Sex	Female	Male	Male	Male	Female	Female
Age (years)	60	50	50	55	54	61
BMI (kg/m^2^)	21.08	29.38	27.64	24.51	21.48	21.63
Basic liver disease	No	HCV	No	HCV	No	No
Tumor size (cm)	–	1.5	5.0	7.3	9.9	5.0
Tumor number	–	1	1	1	1	1
PVTT	No	No	No	Yes	No	No
BCLC stage	–	A	–	C	–	–
ASA score	1	2	1	2	1	2
Prior abdominal history	No	No	Yes	No	No	No
Cirrhosis	No	Yes	No	Yes	No	No
Preoperative diagnosis	Extra- and intrahepatic cholangiolithiasis	HCC	Hemangiomas	HCC	Hemangiomas	ICC
HB (g/L)	126	164	146	158	120	102
PLT ( × 10^9^/L)	128	70	137	117	204	259
PT (s)	12.7	12.2	13.3	14.0	12.7	13.2
AST (U/L)	15	32	27	47	21	32
ALT (U/L)	13	26	32	32	13	30
Albumin (g/L)	39.9	43.4	39.7	35.5	40.1	39.9
Total bilirubin (μmol/L)	15.60	4.54	5.60	10.98	14.30	18.20
AFP (ng/ml)	–	39.20	–	19,264	–	0.97
DCP (mAU/ml)	–	42.8	–	25,683	–	24.36
CEA (ng/ml)	–	0.91	–	1.92	–	0.68
CA19-9 (U/ml)	–	16.45	–	25.43	–	121.4
HCV RNA (IU/ml)	–	Undetected	–	1.37∗10^2^	–	–
Child-Pugh class	A	A	A	A	A	A

BMI, body mass index; PVTT, portal vein tumor thrombus; BCLC, Barcelona clinic liver cancer; ASA score, American Society of Anesthesiologists Score; HB, Hemoglobin; PLT, platelet; PT, prothrombin time; AST, aspartate aminotransferase; ALT, alanine aminotransferase; AFP, alpha-fetoprotein; DCP, des-gamma-carboxy prothrombin; CEA, carcinoembryonic antigen; CA19-9, carbohydrate antigen 19-9; HCV, hepatitis C virus; HCC, hepatocellular carcinoma; ICC, intrahepatic cholangiocarcinoma.

### Intraoperative data of patients

All procedures were successfully completed without conversion to open surgery or local treatment. Detailed perioperative parameters, including operation date, surgical approach, total operative time, robotic system docking duration, console operation time, intraoperative blood loss, and transfusion requirements, were systematically documented in Table 2.

**Table 2:** Intraoperative data of patients.

Parameters	Patient 1	Patient 2	Patient 3	Patient 4	Patient 5	Patient 6
Conversion	No	No	No	No	No	No
R0 resection	–	Yes	–	Yes	–	Yes
Operation	Left hemihepatectomy	Left lateral lobectomy	Left lateral lobectomy	Left hemihepatectomy	Left hemihepatectomy + partial resection of segment 8	Segmentectomy of S4b + S5
Lymph node dissection	No	No	No	No	No	Yes
Additional operation	Cholecystectomy, Cholangioscopic lithotripsy	No	No	Cholecystectomy	Cholecystectomy	Cholecystectomy
Operative date	2025-03-24	2025-03-26	2025-03-28	2025-04-02	2025-04-16	2025-04-18
Operative time (min)	400	190	155	340	240	280
Surgeon console time (min)	247	85	69	197	117	176
Docking time (time)	22	18	23	16	20	19
Pringle maneuver Duration (min)	38	10	10	55	20	25
Blood loss (ml)	20	5	10	200	20	50
Intraoperative RBCs transfusion (Units)	No	No	No	No	No	No
Intraoperative complication	No	No	No	No	No	No

IVC, inferior vena cava; RBC, red blood cell.

### Postoperative data of patients

**Fig. 1:**
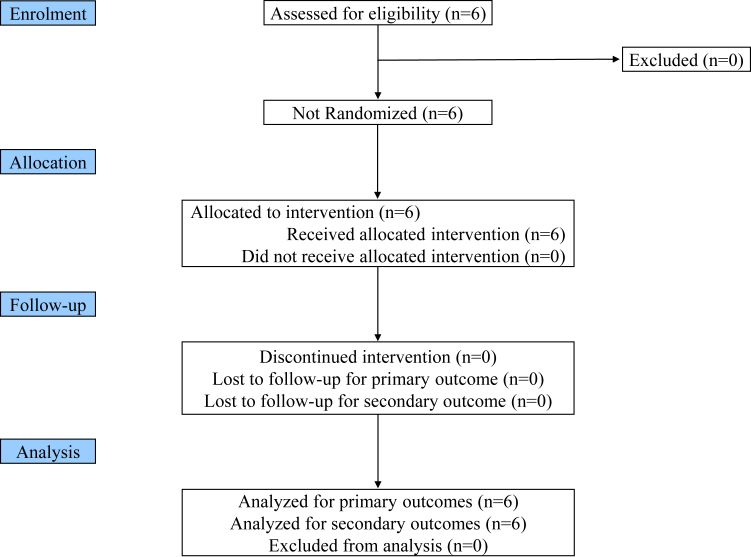
Flow diagram.

All patients achieved uneventful postoperative recovery and were discharged without requiring reoperation. All patients completed follow-up and we got all primary and secondary outcomes as planned (Fig. 1). Notably, the second patient with left lateral lobe experienced bacteremia within 30 days postoperatively, necessitating readmission. This complication was successfully managed with antibiotic therapy, leading to subsequent discharge. Detailed postoperative outcomes including hospital stay duration, complication profiles, and pathological parameters are systematically presented in Table 3.

**Table 3:** Postoperative data of patients.

Parameters	Patient 1	Patient 2	Patient 3	Patient 4	Patient 5	Patient 6
**Postoperative hospital stay (days)**	10	8	5	5	8	9
**Complication**	No	Bacteremia	No	No	No	No
**Complication treatment**	–	Antibiotics	–	–	–	–
**Clavien-Dindo grade**	–	Ⅱ	–	–	–	–
**30-day readmission**	No	Yes	No	No	No	No
**Reoperation**	No	No	No	No	No	No
**Pathologic information**						
Histopathology	Cholelithiasis	ICC	Hemangiomas	HCC	Hemangiomas	ICC
Differentiation grade	–	Median	–	Median	–	Median
Microvascular invasion	–	No	–	Yes	–	Yes
Satellites	–	No	–	No	–	No
Lymph node metastasis	–	–	–	–	–	No
**TOLS**	Yes	No	Yes	Yes	Yes	Yes

TOLS, textbook outcome in liver surgery; HCC, hepatocellular carcinoma; ICC, intrahepatic cholangiocarcinoma.

### Evaluation of telesurgery

**Fig. 2:**
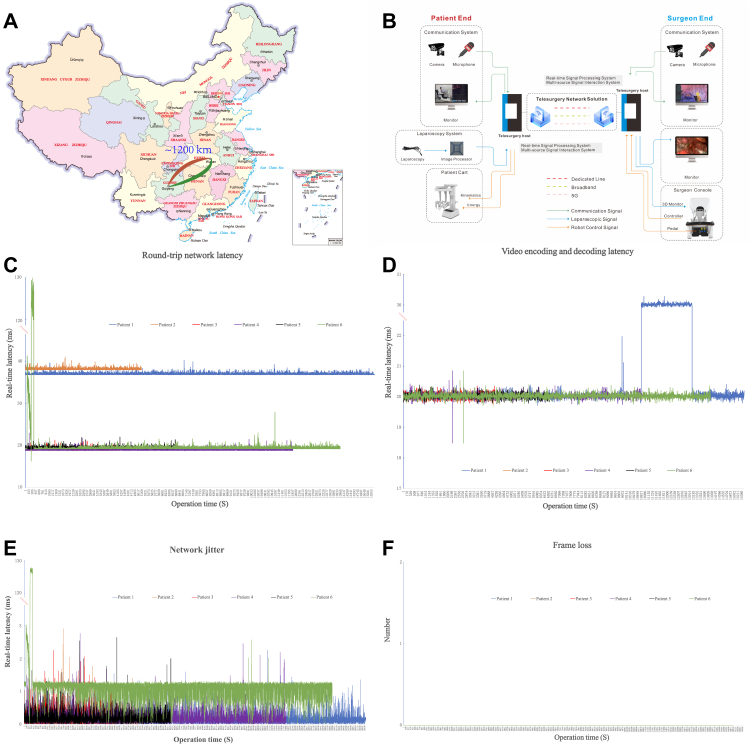
Network connection of remote liver resection. (A) Two hospitals are located in Wuhan, Hubei and Guiyang, Guizhou, respectively. The one-way communication distance is approximately 1200 km. (B) Schematic diagram of telesurgery system. It includes a remote console in Wuhan, and a robotic arm system and a spare console in Guiyang. An AAA-grade optical transport network (OTN) dedicated line is employed to perform the communication task between the two hospitals. The telemedicine system uses 5G wireless network to facilitate real-time communication and live intraoperative imaging between the primary surgeon and assistants. (C) The real-time round-trip network latency was between 16.20 and 129.58 ms. (D) The video encoding and decoding latency was between 18.48 and 30.29 ms. (E) Most of the time, the network jitter was <1 ms except for patient 6, which reached as high as 128.53 ms. (F) Frame loss event was not observed.

**Fig. 3:**
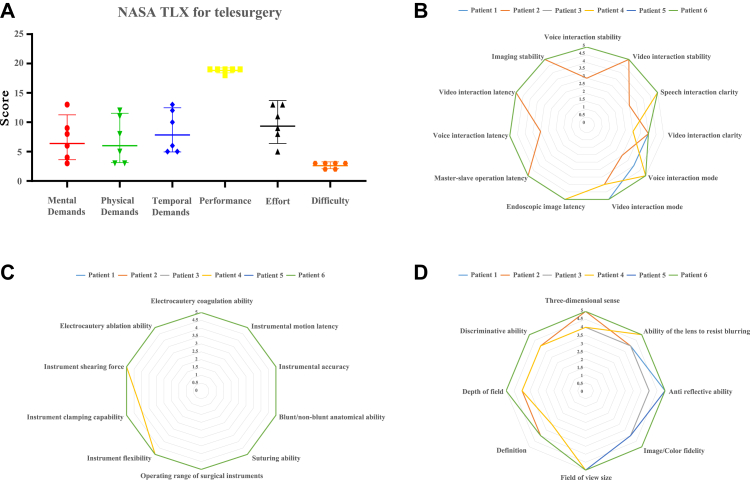
Subjective evaluation of the telesurgery. (A) NASA Task Load Index (Subjective evaluation, Score 1–21, mean value and 95% confidence interval). (B) Latency and stability evaluation of the telesurgery system. (C) Operational stability evaluation of the telesurgery system. (D) Endoscopic image evaluation of the telesurgery system. (B)–(D): Score 1–5. The number of the enrolled patients is 6. Score 1: Very unsatisfied, completely unable to operate; Score 2: Quite unsatisfied, unable to meet the minimum clinical needs; Score 3: basically satisfied, meeting the minimum clinical needs; Score 4: Quite satisfied, meeting most clinical needs; Score 5: Very satisfied, meeting all clinical needs.

Except for Patient 6, whose surgery was temporarily interrupted for approximately 3 min due to a disconnection in the network cable, no other network-related incidents occurred, ensuring the smooth completion of all procedures. As shown in Fig. 2A and B, the two hospitals involved in this study were located in Wuhan and Guiyang, with a one-way communication distance of 1200 km between them. Wuhan was equipped with a remote console, while Guiyang housed a complete surgical robotic system, including both the console and robotic arm. The telecommunication network for the robotic system utilized an AAA-grade optical transport network (OTN) dedicated line with a bandwidth of 100 Mbps, characterized by ultra-low latency, high reliability, and robust security. The telemedicine system employed 5G wireless connectivity to facilitate real-time communication between the primary surgeon and assistants, as well as live transmission of intraoperative imaging. Key intraoperative telecommunication metrics—including round-trip network latency, network jitter, video encoding/decoding latency, and frame loss—were meticulously recorded and are presented in Fig. 2(C–F). After rigorous preoperative preparation, all surgeries were successfully completed without complications. The overall performance of the remote system met the requirements for telesurgery. Detailed assessments of surgeon workload, as well as evaluations of video quality, audio clarity, and instrument functionality for each case, are summarized in Fig. 3.

## Discussion

Telesurgery represents a core strength of robotic surgery and aligns with one of the original purposes behind its invention. Given the high risks of liver resections—especially uncontrolled bleeding—assessing remote surgery's feasibility, safety, and preliminary guidelines is critical.^1^^,^^14^^,^^16^ This pioneering animal experiment performed by Professor Liu initially affirmed the viability of domestic robotic 5G-assisted remote liver resection.^9^ However, it took another five years before the research team headed by Professors Cai Xiujun and Liang Xiao presented a clinical case of robotic remote left lateral lobe resection for the treatment of hepatic hemangioma.^17^ Although isolated cases of remote liver resection have been sporadically reported in the media, the absence of consecutive cohort studies has made it challenging to comprehensively evaluate its feasibility and efficacy. Consequently, we have designed this small-scale prospective single-arm cohort study. To the best of our knowledge, this is also the first report of a consecutive remote liver resection cohort.

In a 1.5-month study, we successfully performed six remote hepatectomies using the MP1000 system (2 left lateral lobectomies, 3 left hemihepatectomies [1 with bile duct exploration, 1 with segment 8 resection], and 1 segments 4b/5 resection with lymph node dissection) via 5G networks. All patients recovered well, demonstrating preliminary feasibility and safety of remote complex liver surgery. Key findings follow:

First and foremost, rigorous preoperative evaluation is paramount. This encompasses two critical aspects: (1) Surgical team preparation and (2) patient preoperative preparation. The surgical team must overcome the learning curve.^1^ Each patient requires preoperative 3D reconstruction and structured MDT discussions, with emphasis on team communication to preemptively address surgical risks.^18^ This approach proved invaluable in several challenging cases: (1) Rh-negative blood type patients required meticulous perioperative planning; (2) successful navigation of anatomical variation where the right posterior bile duct drained anomalously into the left hepatic duct during a left hemihepatectomy; and (3) ligated the origin of left portal vein in one patient with tumor thrombus. These cases highlight how meticulous preoperative planning ensures safety and success in 5G-enabled remote complex hepatectomies.

Second, a stable and high-speed network connection is the fundamental prerequisite for the success of remote surgery.^18^^,^^19^ Before robotic docking, rigorous bandwidth and latency testing is essential to ensure stable connectivity—critical for surgeries like hepatic resections—while balancing image quality and compression efficiency remains challenging.^18^^,^^20^ However, optimized remote surgical image remains functionally adequate yet still falls short of modern high-definition standards.^21^ This technical limitation underscores the need for continued innovation and refinement in remote imaging technologies. During the procedure, dedicated network engineers must continuously monitor signal integrity and system performance. During the sixth case's hilar lymphadenectomy, an anesthesiologist inadvertently displaced the network equipment, resulting in a sudden physical disconnection. The system immediately triggered an alarm, prompting swift action. The on-site surgical team continuously monitored the abdominal cavity, retracted all instruments to avoid inadvertent injury, and maintained readiness to take over immediately if needed. Network engineers from both ends promptly established a video call via mobile communication software, identified the cause of interruption, and restored the link rapidly. Fortunately, through the coordinated efforts of on-site and remote teams, connectivity was restored within 4 min, allowing the surgery to continue without compromising patient safety. Our findings suggest that future standardized emergency protocols should prioritize: On-site backup team with emergency surgical competency; Dedicated network engineers at both operative sites; Redundant network connections to enhance fault tolerance if possible.

Third, intraoperative precision is absolutely critical in remote surgery, particularly during hepatic parenchymal transection—the most hemodynamically challenging phase of liver resection where most bleeding typically arises from the hepatic venous system due to the routine application of the Pringle maneuver.^22^ To address these risks, surgeons must use ultrasonic scalpels in small, controlled steps to maintain optimal visualization and tactile feedback to avoid unintended injuries.^23^^,^^24^ Surgeons can significantly reduce intraoperative hazards while maintaining both patient safety and procedural efficiency by meticulously adhering to these safe principles.

Fourth, the console surgeon must maintain uninterrupted video contact with the remote team, mitigating spatial disconnection through real-time immersion akin to local robotic surgery. Simultaneously, seamless and immediate communication with the team members is paramount to safeguarding patient safety throughout the procedure.^18^ Moreover, the surgeon's real-time interpretation of ultrasonic scalpel auditory cues improves tactile control, enabling precise hepatic dissection and vascular management. This multi-sensory integration of visual, auditory, and communicative feedback is fundamental to achieving optimal outcomes in remote hepatectomy.

The safety assurance and associated ethical considerations of remote surgery remain subjects of intense and ongoing debate.^3^ These discussions fundamentally reflect broader concerns about the efficacy and safety of this emerging surgical modality. In our study, key ethical considerations include: (1) complete disclosure of robotic surgery risks to patients, (2) robust protection of medical data privacy, and (3) clear delineation of responsibilities between surgeons and robotics companies to safeguard treatment quality and patient safety.

This study has several limitations that warrant acknowledgment. This single-arm study, while lacking a conventional robotic surgery control group and having limited sample size, met regulatory sample requirements and upheld rigorous ethical standards. Future research will expand to cohorts and include comparative analyses. These investigations will systematically evaluate both safety outcomes and clinical efficacy, aiming to establish more robust evidence for this innovative surgical approach.

Remote liver resection represents a feasible and effective minimally invasive modality for managing both benign and malignant hepatic pathologies. Further validation through multicenter, large-scale randomized controlled trials (RCTs) remains essential to substantiate these preliminary findings, establish standardized protocols, and ultimately advance the adoption of remote liver resection as a mainstream therapeutic option in clinical practice.

## Contributors

Wei Liao and Hai-Tao Zhu contributed equally to this work. Conceptualization: Wei Liao, Xiao-Ping Chen, Peng Zhu; Accessed and verified the underlying data: Wei Liao, Peng Zhu; Formal analysis: Wei Liao, Gen Cheng, Nan Qiao, Peng Zhu; Investigation: Wei Liao, Hai-Tao Zhu, Yu-Ting Guo, Gen Cheng, Nan Qiao, Peng Zhu; Methodology: Bi-Xiang Zhang, Xiao-Ping Chen, Peng Zhu; Project administration: Xiao-Ping Chen, Peng Zhu; Resources: Hai-Tao Zhu, Yu-Ting, Xiao-Ping Chen, Peng Zhu; Software: Gen Cheng, Nan Qiao; Supervision: Xiao-Ping Chen, Peng Zhu; Validation: Wei Liao, Hai-Tao Zhu, Yu-Ting Guo, Peng Zhu; Writing: Wei Liao, Bi-Xiang Zhang, Xiao-Ping Chen, Peng Zhu. All authors reviewed the manuscript, approved the submitted version, and had final responsibility for the decision to submit it for publication.

## Data sharing statement

The data that supports the findings of the study is available from the corresponding author (Peng Zhu) upon reasonable request.

## Declaration of interests

All authors declare no competing interests.

## Appendix A Supplementary data

Clinical Trial Protocol

Supplementary scale records

Scoring table of telesurgery Chinese-English

Translated Abstract
