# Supplementary material for: Feasibility and safety of remote robotic hepatectomy: a prospective single-arm study with MP1000 system in China
Source: eClinicalMedicine. 2025 Oct 22;89:103579. doi: 10.1016/j.eclinm.2025.103579 (PMC12589949; doi:10.1016/j.eclinm.2025.103579)

医疗器械临床试验方案
Medical Device Clinical Trial Protocol

方案编号：JF-CTP-MP1000-006
Protocol No.: JF-CTP-MP1000-006

评价胸腹腔内窥镜手术系统（MP1000）远程肝脏手术安全性和有效性的临床试验
Clinical trial to evaluate the safety and efficacy of MP1000 system in remote liver surgery

试验医疗器械名称：胸腹腔内窥镜手术系统
Name of test medical device: Thoracic and Abdominal Endoscopic Surgery System
型号规格：MP1000
Model and specification: MP1000
需进行临床试验审批的第三类医疗器械 是□ 否☒
方案版本号和日期：V01，2024 年 09 月 30 日 临床试验机构：1.华中科技大学同济医学院附属同济医院
For Class III medical devices requiring clinical trial approval, protocol version number and date: V01, September 30, 2024 Clinical trial institution: 1. Tongji Hospital Affiliated to Tongji Medical College, Huazhong University of Science and Technology
2.贵州医科大学附属医院
协调研究者：陈孝平
2. Coordinating researcher of Affiliated Hospital of Guizhou Medical University: Chen Xiaoping
申办者：深圳市精锋医疗科技股份有限公司
Sponsor: Shenzhen Jingfeng Medical Technology Co., Ltd.

填写说明
Fill in the Instructions

1.申办者应当根据试验目的，综合考虑试验医疗器械的风险、技术特征、适用范围等，组织制定科学、合理的临床试验方案。

The sponsor shall organize and formulate a scientific and reasonable clinical trial plan according to the purpose of the trial, taking into account the risks, technical characteristics and scope of application of the trial medical devices.

2.本方案应当由主要研究者签名和注明日期，经医疗器械临床试验机构审核签章后交申办者。

The protocol shall be signed and dated by the principal investigator, and submitted to the sponsor after being reviewed and sealed by the medical device clinical trial institution.

3.可附方案历次修订情况以及理由。
The previous revisions of the scheme and the reasons can be attached.

4.方案应当有目录。
The programme should have a catalogue.

5.可根据需要增加缩略语表、参考文献等内容。
Abbreviations and references can be added as required.

方案版本修订记录
Revision record of scheme version

版本<br>Version	日期<br>Date	原因<br>Reason	修订内容<br>Revised content
V01	2024年09月30日<br>September 30, 2024	初始版本<br>Initial version	/

目录
Catalog

目录 4
Catalog 4
方案摘要 6
Summary of the programme 6
一、申办者信息 8
I. Information of the Applicant 8
（一）申办者名称 8
(I) Name of the applicant 8
（二）申办者地址 8
(2) Address of the applicant 8
（三）申办者联系方式 8
(III) Contact information of the applicant 8
二、临床试验机构和主要研究者信息 8
II. Information of Clinical Trial Institution and Principal Investigator 8
三、临床试验的背景资料 8
III. Background information of clinical trial 8
（一）研发背景 8
(I) R & D background 8
（二）产品基本信息（包括结构组成、工作原理、作用机理、产品特点等） 10
(2) Basic information of the product (including structural composition, working principle, mechanism of action, product characteristics, etc.) 10
1. 产品结构组成 10
Product structure composition 10
2. 工作原理 10
How it works 10
3. 远程手术应急预案 12
Telesurgery contingency plan 12
（三）适用范围以及相关信息 12
(3) Scope of application and relevant information 12
四、试验目的 12
IV. Test Purpose 12
五、试验设计 12
V. Test design 12
（一）总体设计以及确定依据 13
(I) Overall design and basis for determination 13
（二）受试者选择 13
(II) Subject selection 13
1. 入选标准 13
Inclusion criteria 13
2. 排除标准 13
Exclusion criteria 13
3. 停止试验的标准和程序 14
Criteria and procedures for stopping the test 14
（三）评价方法 14
(III) Evaluation method 14
1. 主要评价指标 14
Main evaluation indicators 14
2. 次要评价指标 15
Secondary evaluation index 15
3. 整机安全性评价和不良事件、严重不良事件 16
Safety evaluation of the whole machine, adverse events and serious adverse events 16
（四）试验医疗器械和对照医疗器械 17
(IV) Test medical devices and control medical devices 17
1. 试验医疗器械 17
Test medical device 17
2. 对照医疗器械 18
Control medical device 18
（五）试验流程 18
(V) Test process 18
1. 试验流程表 18
Test flow chart 18
2. 试验实施（方法、内容、步骤等） 20
Test implementation (methods, contents, steps, etc.) 20
3. 用械规范 20
Equipment specification 20
4. 合并用药规范 21
Code of practice for concomitant medication 21
（六）偏倚控制措施 21
(VI) Bias control measures 21
六、统计学考虑 22
VI. Statistical considerations 22
（一）样本量估算 22
(I) Sample size estimation 22
1. 样本量 22
Sample size 22

2. 样本量分配以及其确定依据 22
Sample size allocation and the basis for its determination 22
（二）分析数据集 22
(II) Analytical data set 22
（三）受试者剔除标准 23
(III) Subject exclusion criteria 23
（四）统计方法 23
(4) Statistical methods 23
1. 统计分析原则 23
Principles of statistical analysis 23
2. 完成情况及人口学分析 23
Completion and Demographic Analysis 23
3. 主要评价指标 23
Main evaluation indicators 23
4. 次要评价指标 24
Secondary evaluation index 24
5. 整机安全性评价和不良事件、严重不良事件 24
Safety evaluation of the whole machine, adverse events and serious adverse events 24
（五）缺失值和异常值的处理 24
(V) Handling of missing values and abnormal values 24
1. 缺失值处理 24
Missing value processing 24
2. 不合理数据处理 24
Unreasonable data processing 24
3. 错误数据处理 24
Error data processing 24
七、监查计划 25
VII. Audit Plan 25
八、数据管理 26
VIII. Data Management 26
（一）EDC 数据管理 26
(I) EDC data management 26
（二）记录保存 27
(II) Record keeping 27
九、风险受益分析 27
IX. Risk and Benefit Analysis 27
十、临床试验的质量控制 27
X. Quality Control of Clinical Trial 27
十一、临床试验的伦理问题以及知情同意 28
Ethical Issues in Clinical Trials and Informed Consent 28
（一）伦理方面的考虑 28
(I) Ethical considerations 28
（二）知情同意过程 28
(II) Informed consent process 28
十二、对不良事件和器械缺陷报告的规定 28
XII. Provisions on Adverse Event and Device Defect Reporting 28
（一）不良事件的定义和报告规定 28
(I) Definition and reporting requirements of adverse events 28
（二）器械缺陷 29
(II) Device defect 29
（三）严重不良事件的定义 29
(III) Definition of serious adverse event 29
（四）报告程序、联络人信息 29
(4) Information on reporting procedures and contact persons 29
1. 研究者 29
Researcher 29
2. 申办者 30
Sponsor 30
3. 联络人信息 30
Contact information 30
十三、临床试验方案的偏离与临床试验方案修正的规定 31
XIII. Provisions on deviation from and amendment to the clinical trial protocol 31
（一）临床试验方案的偏离 31
(I) Deviation from clinical trial protocol 31
（二）临床试验方案的修正 31
(II) Amendment of clinical trial protocol 31
十四、直接访问源数据、文件 31
14. Direct access to source data and files 31
十五、临床试验报告应当涵盖的内容 32
XV. Contents to be covered in the clinical trial report 32
十六、保密原则 32
XVI. Confidentiality 32
十七、各方承担的职责 32
XVII. Responsibilities of the Parties 32
（一）申办者职责 33
(I) Responsibilities of the Applicant 33
（二）临床试验机构和研究者职责 35
(II) Responsibilities of clinical trial institutions and researchers 35

附录 1 术后 Clavien-Dindo 系统分级 39
Appendix 1 Grading of Clavien-Dindo System after Operation 39
附录 2 视觉模拟评分表（Visual Analogue Scale/Score VAS） 40
Appendix 2 Visual Analogue Scale (Visual Analogue Scale/Score VAS) 40
附录 3 NASA-TLX 量化表 41
Appendix 3 NASA-TLX Quantification Table 41
附录 4 远程手术操作评分表 42
Appendix 4 Score Sheet of Remote Surgical Operation 42
附录 5 远程手术网络传输记录表 43
Appendix 5 Remote Surgery Network Transmission Record 43
研究者声明 44
Investigator statement 44

方案摘要
Summary of the Programme

**试验名称**<br>Test name	评价胸腹腔内窥镜手术系统（MP1000）远程肝脏手术安全性 和有效性的临床试验<br>Clinical trial to evaluate the safety and efficacy of MP1000 system in remote liver surgery
**申办者**<br>Sponsor	深圳市精锋医疗科技股份有限公司<br>Shenzhen Jingfeng Medical Technology Co., Ltd.
**器械分类**<br>Device classification	III类<br>Class III
**试验设计**<br>Experimental design	前瞻性、多中心、单组<br>Prospective, multicenter, single-arm
**试验目的**<br>Purpose of the test	通过本临床试验，评价和验证深圳市精锋医疗科技股份有限公司生产的胸腹腔内窥镜手术系统（MP1000）远程手术的安 全性和有效性。<br>Through this clinical trial, evaluate and verify the safety and effectiveness of the thoracoabdominal endoscopic surgery system (MP1000) produced by Shenzhen Jingfeng Medical Technology Co., Ltd.
**样本量**<br>Sample size	6 例<br>6 cases
**适用范围**<br>Scope of application	该产品由医师利用主从操控系统对于微创手术器械进行控制，用于泌尿外科、妇科、普通外科和胸外科手术。荧光成像功能用于提供手术部位的可见光及近红外光成像，其中近红外光成像需配合已在中国批准上市且应用部位一致的吲哚菁绿使用。远程操作功能用于提供微创手术器械的远程控 制，实现远程手术操作。<br>The product is controlled by physicians using master-slave control system for minimally invasive surgical instruments, which are used in urology, gynecology, general surgery and thoracic surgery. The fluorescence imaging function is used to provide visible light and near-infrared light imaging of the surgical site, in which the near-infrared light imaging needs to be used in conjunction with indocyanine green, which has been approved for marketing in China and has the same application site. The remote operation function is used to provide remote control of minimally invasive surgical instruments and realize remote surgical operation.
**入选标准**<br>Inclusion criteria	可以加入本研究的受试者，必须符合下述所有条件：<br>Subjects who may be enrolled in the study must meet all of the following conditions:<br>1）18-80 周岁（含），男女不限；<br>1) 18-80 years old (inclusive), male or female;<br>2）BMI：18-30Kg/m^2；<br>3） 有肝脏手术的相关指征；<br>Indications for liver surgery;<br>4） 生理状况可接受腹腔镜手术者；<br>Physiologically acceptable for laparoscopic surgery;<br>5） 愿意配合并完成研究随访及相关检查；<br>Willing to cooperate and complete the study follow-up and related examinations;<br>6） 自愿签署知情同意书者。<br>Those who voluntarily sign the informed consent form.
**排除标准**<br>Exclusion criteria	如遇下列任何情况之一，受试者不应参加试验：<br>Subjects should not participate in the trial if any of the following conditions occur:<br>1） 有严重心血管或循环系统疾病且不能耐受手术；<br>Have severe cardiovascular or circulatory disease and cannot tolerate surgery;<br>2） 妊娠或哺乳；<br>Pregnancy or lactation;

	3）有癫痫或精神病史；<br>History of epilepsy or mental illness;<br>4）严重过敏体质和疑似或已确诊的酒精或药物成瘾；<br>Severe allergies and suspected or confirmed alcohol or drug addiction;<br>5）无法理解研究要求，或不能完成研究随访计划；<br>Inability to understand the study requirements or to complete the study follow-up plan;<br>6）研究者认为不适宜参加本试验者。<br>Those who are not considered suitable to participate in this trial by the investigator.
**主要评价指标**<br>Main evaluation indicators	主要有效性评价指标：手术成功率<br>Main effectiveness evaluation index: success rate of operation<br>主要安全性评价指标：3 级或以上并发症发生率<br>Main safety evaluation index: Incidence rate of grade 3 or above complications
**次要评价指标**<br>Secondary evaluation index	1) 整体并发症发生率<br>Overall complication rate<br>2) 手术时间<br>Time of operation<br>3) 医生控制台操作时间<br>Doctor Console Operation Time<br>4) 术中估计失血量<br>Estimated intraoperative blood loss<br>5) 切缘阳性率<br>Positive rate of incisal margin<br>6) 患者疼痛评分<br>Patient pain score<br>7) 医生满意度<br>Doctor satisfaction<br>8) 术后住院时间<br>Postoperative hospital stay<br>9) 输血率<br>Blood transfusion rate<br>10) 再入院率<br>Readmission rate<br>11) 再次手术率<br>Reoperation rate<br>12) 死亡率<br>Mortality<br>13) 器械缺陷发生率<br>Incidence of device defects<br>14) 不良事件发生率<br>Incidence of adverse events<br>15) 严重不良事件发生率<br>Incidence of serious adverse events<br>16) 远程操作评价<br>Remote operation evaluation
**观察周期**<br>Observation period	30 天<br>30 days
**统计分析**	具体统计分析内容见统计分析计划

Statistical analysis	See the statistical analysis plan for specific statistical analysis contents.
**试验周期**<br>Test period	12 个月<br>12 months

一、申办者信息
I. Information of the Applicant

（一）申办者名称
(I) Name of the Applicant

深圳市精锋医疗科技股份有限公司

Shenzhen Jingfeng Medical Technology Co., Ltd.

（二）申办者地址
(2) Address of the Applicant

深圳市龙岗区宝龙街道宝龙社区宝荷大道 76 号智慧家园二期 2B1901

2B1901, Wisdom Home Phase II, No.76, Baohe Avenue, Baolong Community, Baolong Street, Longgang District, Shenzhen City

（三）申办者联系方式
(III) Contact Information of the Applicant

联系人：毛建乐

Contact: Mao Jianle

联系电话：13823125005

Contact number: 13823125005.

二、临床试验机构和主要研究者信息
II. Information of Clinical Trial Institution and Principal Investigator

临床试验 Clinical trials 机构代号 Organization code	临床试验机构名称 Name of clinical trial institution	主要研究者 Principal Investigator	科室 Department	职称 Title
01	华中科技大学同济医学院附属 Affiliated to Tongji Medical College of Huazhong University of Science and Technology 同济医院 Tongji Hospital	陈孝平 Chen Xiaoping	肝脏外科 Liver surgery	主任医师 Chief physician
02	贵州医科大学附属医院 Affiliated Hospital of Guizhou Medical University	李海洋 Li Haiyang	肝胆外科 Hepatobiliary surgery	主任医师 Chief physician

注：临床试验过程中，根据实际情况进行临床参与单位的增减，届时将不进行方案修正，但新增的临床单位应当完成方案签署页。

Note: During the clinical trial, the number of clinical participating units will be increased or decreased according to the actual situation, and the protocol will not be amended at that time, but the newly added clinical units shall complete the signature page of the protocol.

三、临床试验的背景资料
III. Background Information of Clinical Trial

（一）研发背景
(I) R & D Background

作为一种重要的医学手段，近年来外科手术技术取得了长足的进展，已经由最初的开放手术及其后大规模应用的微创腔镜手术，发展到目前正迅速推广的微创腔镜机器人手术的阶段。目前腔镜机器人已经应用于临床 20 余年，得到了广泛应用并获得了良好的效果。

As an important medical means, surgical technology has made great progress in recent years, which has developed from the initial open surgery and the subsequent large-scale application of minimally invasive endoscopic surgery to the current stage of rapidly promoting minimally invasive endoscopic robotic surgery. At present, endoscopic robot has been used in clinic for more than 20 years, and has been widely used and achieved good results.

相比传统的腔镜手术，机器人进行腔镜手术有许多优势：

Compared with traditional endoscopic surgery, robotic endoscopic surgery has many advantages:

1)提供主刀医生高清晰、立体的手术视野，符合人类工程学，让医生拥有和人眼一样的立体式感觉，可以清晰准确地进行组织定位和器械操作；

Provide the surgeon with a high-definition, three-dimensional surgical field of vision, in line with ergonomics, so that doctors have the same three-dimensional feeling as the human eye, can clearly and accurately carry out tissue positioning and instrument operation;

2)手术器械可以模拟人的手指的灵活度，同时消除不必要的颤动，所以手术器械

The surgical instrument can simulate the flexibility of human fingers and eliminate unnecessary vibration, so the surgical instrument

完全达到人手的灵活度和准确度，可以进行人手不能触及的狭小空间的精细手术操作；
Fully achieve the flexibility and accuracy of human hands, and can perform fine surgical operations in a narrow space which cannot be touched by human hands;

3)医生使用含手脚操作装置的医生控制台，操控精密机器臂及立体内窥镜，操作方式完全尊重医生开放手术操作方式，不需要长时间的培训和学习；
The doctor uses a doctor console containing a hand and foot operation device to control a precision robot arm and a three-dimensional endoscope, and the operation mode fully respects the doctor's open operation mode without long-term training and learning;

4)主刀医生可以同时控制内窥镜和手术器械，减少了对其他手术团队成员的配合需求，更容易实现主刀医生的意图；
The surgeon can control the endoscope and surgical instruments at the same time, which reduces the cooperation requirements of other surgical team members and makes it easier to realize the intention of the surgeon.

5)主刀医生采取坐姿进行系统操作，舒适的坐姿有利于长时间复杂的手术，而且延长了主刀医生的手术生命；
The chief surgeon adopts the sitting posture to carry out the systematic operation, and the comfortable sitting posture is beneficial to the long-time complicated operation and prolongs the operation life of the chief surgeon;

6)患者从这种新手术方式受益良多，切口变小、康复时间缩短、住院天数减少。在对大多手术而言，复原时间大幅缩短，患者可快速地恢复日常作息。减少麻醉需求量、感染风险、失血量或输血必要、创伤和疤痕，减少术中组织创伤炎性反应导致的术后粘连，术后预后更加良好。
Patients benefit a lot from this new surgical method, with smaller incisions, shorter recovery time and fewer hospital days. For most operations, the recovery time is greatly reduced and patients can quickly return to their daily routines. Reduce the need for anesthesia, the risk of infection, blood loss or the need for blood transfusion, trauma and scars, reduce postoperative adhesions caused by inflammatory reaction of tissue trauma during operation, and have a better prognosis after operation.

深圳市精锋医疗科技股份有限公司解决了腔镜手术机器人的诸多核心技术（如主从控制策略、运动学研究、多自由度仿真机械手和立体视觉等），解决了腔镜手术机器人的关键技术。并于 2020 年委托北京市医疗器械检验所完成了产品性能检验和安全检验，产品符合国家标准和行业标准。

Shenzhen Jingfeng Medical Technology Co., Ltd. has solved many core technologies of endoscopic surgical robots (such as master-slave control strategy, kinematics research, multi-degree-of-freedom simulation manipulator and stereo vision, etc.), and solved the key technologies of endoscopic surgical robots. In 2020, Beijing Medical Device Inspection Institute was commissioned to complete the product performance inspection and safety inspection, and the products met the national and industrial standards.

深圳市精锋医疗科技股份有限公司生产的胸腹腔内窥镜手术系统（MP1000）已完成泌尿外科、妇科、普外科和胸外科的临床试验，手术成功率 100%，此外不良事件、整机安全性评价、器官和血管损伤事件、实验室检查、生命体征和器械缺陷的评价，充分论证了试验器械满足临床外科手术的有效性和安全性。目前胸腹腔内窥镜手术系统（MP1000）已获得国家药品监督管理局（NMPA）上市批准（注册证编号：国械注准 20223011623）。

The thoracoabdominal endoscopic surgery system (MP1000) produced by Shenzhen Jingfeng Medical Technology Co., Ltd. has completed clinical trials in urology, gynecology, general surgery and thoracic surgery, with a success rate of 100%. In addition, adverse events, safety evaluation of the whole machine, organ and vascular injury events, laboratory tests, vital signs and device defects have been evaluated. It fully demonstrates the effectiveness and safety of the test instrument to meet the clinical surgical operation. At present, the thoracoabdominal endoscopic surgery system (MP1000) has been approved by the National Medical Products Administration (NMPA) (registration certificate number: 20223011623).

手术机器人技术和远程通信技术的长足发展促进了实时远程外科手术实现的可能，让专家能够从数千公里的距离之外直接控制手术机器人系统进行手术操作，同时几乎没有操作延迟。经过对远程技术进行严格而充分的技术论证、上百例动物试验验证，初步证明了远程机器人手术的可行性、安全性和有效性，并且在国产精锋多孔腔镜手术机器人的技术团队辅助下，由中国人民解放军总医院张旭院士团队主刀，跨越 3000 公里，在北京和海南两地之间成功完成了远程手术机器人辅助下腔静脉后输尿管修复成型手术、肾根治性切除术、肾部分切除术、前列腺根治性切除术、肾上腺肿瘤

The rapid development of surgical robotic technology and remote communication technology has promoted the possibility of real-time remote surgery, enabling experts to directly control surgical robotic systems from thousands of kilometers away, with little delay. After strict and full technical demonstration of remote technology and hundreds of animal experiments, the feasibility, safety and effectiveness of remote robotic surgery were preliminarily proved, and with the assistance of the technical team of the domestic Jingfeng Porous Endoscopic Surgical Robot, the team of Academician Zhang Xu of the General Hospital of the Chinese People's Liberation Army was the main knife, spanning 3000 kilometers. Between Beijing and Hainan, it has successfully completed the operation of repairing and shaping the ureter behind the inferior vena cava, radical nephrectomy, partial nephrectomy, radical prostatectomy and adrenal tumor assisted by remote surgical robot.

切除术等手术，所有手术都顺利完成，手术全程视野清晰、机械臂运行灵活，未出现信号卡顿，证明了远程机器人手术在腔镜手术中的安全性和有效性。精锋远程手术系统采用多种技术保障远程手术的顺利进行，术中几乎没有动作延迟，能够确保远程手术中“手-眼协调”的一致性和患者手术的安全性、有效性。

Resection and other operations, all operations were successfully completed, the whole operation has clear vision, flexible operation of the mechanical arm, and no signal stutter, which proves the safety and effectiveness of remote robotic surgery in endoscopic surgery. Jingfeng Remote Surgery System uses a variety of technologies to ensure the smooth progress of remote surgery, with almost no delay in the operation, which can ensure the consistency of "hand-eye coordination" in remote surgery and the safety and effectiveness of patients' surgery.

远程机器人手术能够减少患者异地求医的难度、缓解地区之间的医疗水平差异、在紧急时能够缓解医疗资源分布不均的问题，促进医疗健康服务的创新供给和医疗资源的开放共享，对缩小各地区医疗资源不平衡、提升基层医疗健康服务能力和普惠水平等多方面具有重要意义。

Telerobotic surgery can reduce the difficulty of patients seeking medical treatment in different places, alleviate the differences in medical level between regions, alleviate the uneven distribution of medical resources in emergencies, promote the innovative supply of medical and health services and the open sharing of medical resources. It is of great significance to narrow the imbalance of medical resources in different regions, improve the ability of primary medical and health services and the level of universal benefits.

（二）产品基本信息（包括结构组成、工作原理、作用机理、产品特点等）

(2) Basic Information of the Product (including Structural Composition, Working Principle, Mechanism of Action, Product Characteristics, Etc.)

1. 产品结构组成

Product Structure Composition

本系统由医生控制台（一个或两个）、患者手术平台、影像系统、远程控制平台、手术器械和附件组成。

The system consists of a doctor console (one or two), a patient operation platform, an image system, a remote control platform, surgical instruments and accessories.

2. 工作原理

How It Works

在腔内手术过程中，专用手术器械和立体内窥镜通过多个器械通道进入人体到达手术区域。

In the process of intracavitary surgery, special surgical instruments and three-dimensional endoscopes enter the human body through a plurality of instrument channels to reach the surgical area.

影像处理平台将立体内窥镜获得的手术区域的图像信号进行运算处理，转换成立体图像信号输出至医生控制台内的立体显示器，转换成平面图像信号输出至影像车上的平面显示器。

The image processing platform carries out operation processing on the image signal of the operation area obtained by the three-dimensional endoscope, converts the image signal into a three-dimensional image signal and outputs the three-dimensional image signal to the three-dimensional display in the doctor console, and converts the three-dimensional image signal into a plane image signal and outputs the plane image signal to the plane display on the image trolley.

位于医生控制台处的外科医生根据手术区域的立体图像，按实际临床需要，手部操作传感器和脚部踩踏脚踏开关模拟实际手术操作。

A surgeon positioned at the doctor console simulates the actual surgical operation by operating the sensor with the hand and stepping on the pedal switch with the foot according to the three-dimensional image of the surgical area and the actual clinical needs.

主从控制系统将医生的动作信号进行运算处理，控制手术器械模拟外科医生的手部动作以及控制与有源手术器械连接的高频发生器的能量激励，最终实现精确控制手术操作的目的。

The master-slave control system calculates and processes the action signal of the surgeon, controls the surgical instrument to simulate the hand action of the surgeon, and controls the energy excitation of the high-frequency generator connected with the active surgical instrument, so as to finally realize the purpose of accurately controlling the surgical operation.

位于患者位置的医生助手根据平面图像以及外科医生的指令，按临床实际需要调整设备姿态、装卸专用手术器械、装卸立体内窥镜以及处理一些非预期突发情况等。

According to the plane image and the surgeon's instructions, the doctor's assistant at the patient's position adjusts the equipment posture, loads and unloads the special surgical instruments, loads and unloads the stereo endoscope, and handles some unexpected emergencies according to the actual clinical needs.

远程手术是通过远程控制平台连接两端的机器人系统，利用专网、5G 或宽带网络传输信号，主刀医生在远程的医生控制台控制本地的患者手术平台为患者开展手术，

Remote surgery is to connect the robot systems at both ends through the remote control platform, use the private network, 5G or broadband network to transmit signals, and the chief surgeon controls the local patient surgery platform at the remote doctor console to carry out surgery for patients.

同时实现本地端和远程端的实时沟通。本地的医生控制台与患者手术平台连接，可以手动获取控制权，也可以在远程异常时自动获取控制权，确保完成手术。

At the same time, it realizes real-time communication between local and remote terminals. The local doctor's console is connected with the patient's operation platform, which can manually obtain the control right or automatically obtain the control right in case of remote abnormality to ensure the completion of the operation.

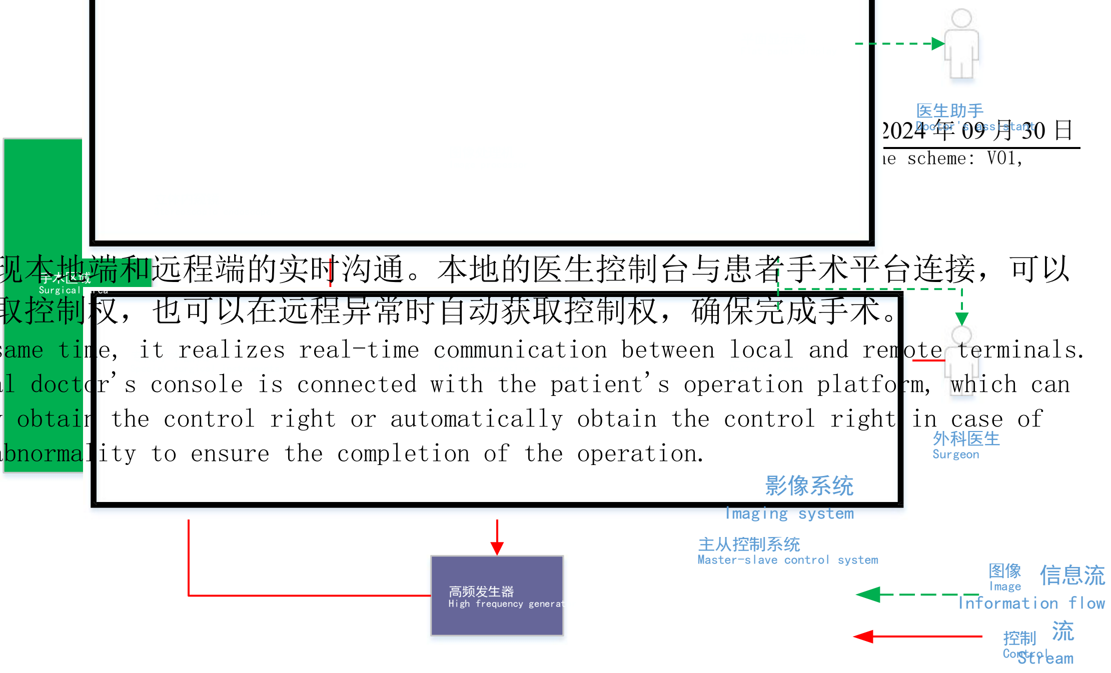

图 1 胸腹腔内窥镜手术系统工作原理示意图

Fig. 1 Schematic diagram of working principle of thoracoabdominal endoscopic surgery system

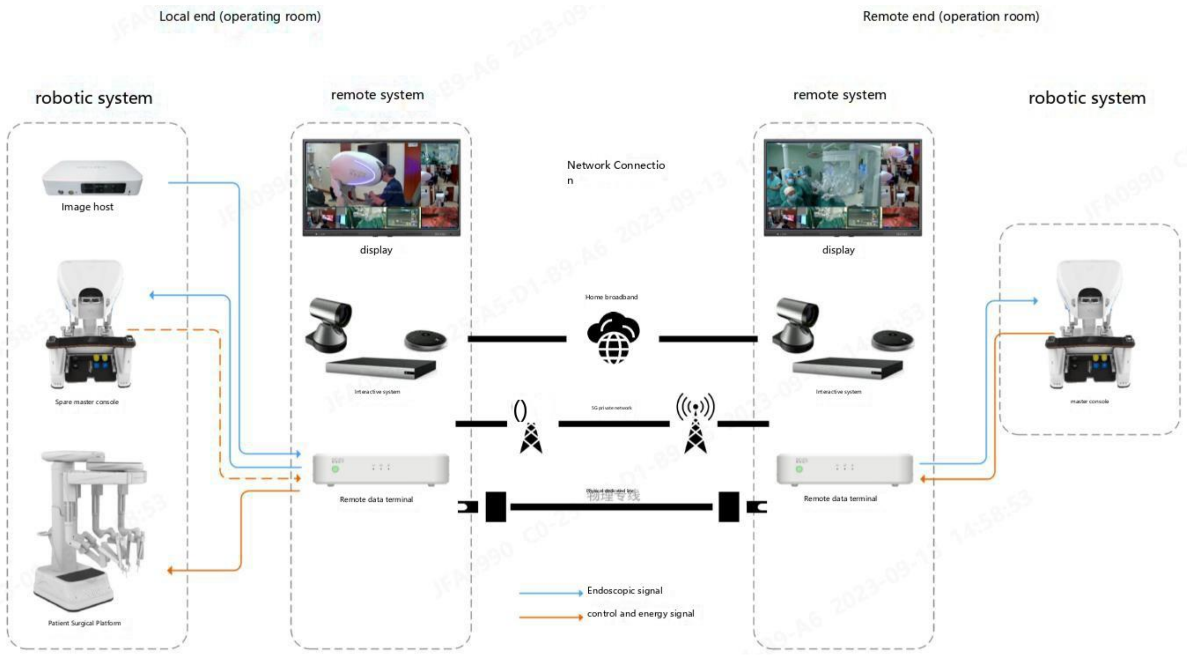

图 2 远程手术工作原理示意图

Figure 2 Schematic diagram of the working principle of remote surgery

3. 远程手术应急预案

Telesurgery Contingency Plan.

1）远程网络异常（包括图像信号异常、控制指令异常等）

Remote network exception (including image signal exception, control instruction exception, etc.)

如果出现远程网络异常且不能及时恢复的情况，应根据现场环境，由患者端现场负责人及时决策是否中转本地手术。

If the remote network is abnormal and cannot be recovered in time, the person in charge of the patient's site should decide whether to transfer to the local operation in time according to the site environment.

2）术中操作异常（包括不能有效止血，精准操作等）

Abnormal operation during the operation (including ineffective hemostasis, accurate operation, etc.)

如果出现网络正常，但是在远程端不能够有效治疗的情况，应根据现场环境，由患者端现场负责人及时决策是否中转本地手术。

If the network is normal, but the remote side can not be effectively treated, the person in charge of the patient side should decide whether to transfer to the local operation in time according to the scene environment.

（三）适用范围以及相关信息

(3) Scope of Application and Relevant Information

1、适应症：该产品由医师利用主从操控系统对于微创手术器械进行控制，用于泌尿外科、妇科、普通外科和胸外科手术。荧光成像功能用于提供手术部位的可见光及近红外光成像，其中近红外光成像需配合已在中国批准上市且应用部位一致的吲哚菁绿使用。远程功能用于提供远程手术操作。

1. Indications: The product is controlled by the master-slave control system for minimally invasive surgical instruments, and is used in urology, gynecology, general surgery and thoracic surgery. The fluorescence imaging function is used to provide visible light and near-infrared light imaging of the surgical site, in which the near-infrared light imaging needs to be used in conjunction with indocyanine green, which has been approved for marketing in China and has the same application site. The remote function is used to provide remote surgical operation.

2、适用人群：没有限制预期治疗的特定人群，由负责腔镜手术的医生依据患者情况来决定是否适用；

2. Applicable population: There is no restriction on the specific population of the intended treatment, and the doctor in charge of endoscopic surgery decides whether it is applicable according to the patient's condition;

3、适用部位：人体的腹腔部位、盆腔部位和胸腔部位；

3. Applicable parts: abdominal cavity, pelvic cavity and thoracic cavity of human body;

4、与人体接触方式和时间：短时间直接接触；

4. Mode and time of contact with human body: direct contact for a short time;

5、疾病的严重程度和阶段：适宜进行腹腔镜、胸腔镜手术期间；

5. Severity and stage of the disease: suitable for laparoscopic and thoracoscopic surgery;

6、使用条件：在医疗机构的手术环境中使用；

6. Conditions of use: use in the surgical environment of medical institutions;

7、重复使用：立体内窥镜、手术器械和附件的使用期限，请参考使用说明书；

7. Reuse: For the service life of stereo endoscope, surgical instruments and accessories, please refer to the instruction manual;

8、使用方法：详见《使用说明书》；

8. Usage: See the User Manual for details;

9、禁忌症：与传统腹腔镜手术禁忌症相同，包括凝血功能严重异常、病态肥胖以及怀孕等。

9. Contraindications: It is the same as the contraindications of traditional laparoscopic surgery, including severe abnormal coagulation function, morbid obesity and pregnancy.

10、警告及预防措施：在产品说明书中对防范措施和安全警告信息进行相关描述。

10. Warning and preventive measure: precautionary measures and safety warning information shall be described in the product manual.

四、试验目的

IV. Test Purpose

通过本临床试验，评价和验证深圳市精锋医疗科技股份有限公司生产的胸腹腔内窥镜手术系统（MP1000）远程手术的安全性和有效性。

Through this clinical trial, evaluate and verify the safety and effectiveness of the thoracoabdominal endoscopic surgery system (MP1000) produced by Shenzhen Jingfeng Medical Technology Co., Ltd.

五、试验设计

V. Test Design

（一）总体设计以及确定依据

(I) Overall Design and Basis for Determination

本次临床试验采用前瞻性、多中心、单组临床试验的设计，选择已经在医疗器械临床试验机构备案管理信息系统备案的医疗器械临床试验机构作为临床试验中心。所有受试者签署知情同意书（Informed consent form，ICF）后，经筛选合格，采集其基本信息，进行临床试验，观察试验器械的安全性和有效性。

This clinical trial adopts the prospective, multi-center and single-group clinical trial design, and selects the medical device clinical trial institution that has been registered in the medical device clinical trial institution registration management information system as the clinical trial center. After all subjects signed the informed consent (Informed consent form, ICF), they were screened to be qualified, and their basic information was collected for clinical trials to observe the safety and effectiveness of the test device.

具体试验方法选择的理由包括：

Reasons for the selection of specific test methods include:

⑴ 前瞻性：采用前瞻性设计不易受偏倚影响，在试验开始之初定好统一的入排标准、试验方法、试验流程及评估标准进行观察，更能保证信息详细可靠。

Prospective: Prospective design is not easy to be affected by bias. At the beginning of the test, the unified inclusion and exclusion criteria, test methods, test procedures and evaluation criteria are set for observation, which can ensure detailed and reliable information.

⑵ 多中心：计划多家中心参与本临床试验。多中心试验可以在较短的时间内搜集所需的病例数，且搜集的病例范围广，其研究结论外推性更好。

Multicenter: Multiple centers are planned to participate in this clinical trial. Multicenter trials can collect the required number of cases in a relatively short period of time, and the range of cases collected is wide, and the extrapolation of research conclusions is better.

⑶ 单组：胸腹腔内窥镜手术系统 MP1000 已进行泌尿外科、妇科、普外科临床试验，并获得注册证，其临床使用的安全性和有效性已得到充分验证。

Single group: MP1000 thoracoabdominal endoscopic surgery system has undergone clinical trials in urology, gynecology and general surgery, and has been registered. Its safety and effectiveness in clinical use have been fully verified.

综合以上考虑，同时基于《医疗器械监督管理条例》、《医疗器械注册与备案管理办法》、《医疗器械临床试验质量管理规范》和《医疗器械临床试验设计指导原则》，本临床试验设计采用前瞻性、多中心、单组临床试验设计。

Based on the above considerations and based on the Regulations on the Supervision and Administration of Medical Devices, the Measures for the Administration of Registration and Filing of Medical Devices, the Good Clinical Practice for Medical Devices and the Guiding Principles for the Design of Clinical Trials of Medical Devices, this clinical trial is designed as a prospective, multi-center and single-group clinical trial.

（二）受试者选择

(II) Subject Selection

1. 入选标准

Inclusion Criteria

可以加入本研究的受试者，必须符合下述所有条件：

Subjects who may be enrolled in the study must meet all of the following conditions:

(1) 18-80 周岁（含），男女不限；
18-80 years old (inclusive), male or female;

(2) BMI：18-30Kg/m^2；

(3) 有肝脏手术的相关指征；
Indications for liver surgery;

(4) 生理状况可接受腹腔镜手术者；
Physiologically acceptable for laparoscopic surgery;

(5) 愿意配合并完成研究随访及相关检查；
Willing to cooperate and complete the study follow-up and related examinations;

(6) 自愿签署知情同意书者。
Those who voluntarily sign the informed consent form.

2. 排除标准

Exclusion Criteria

如遇下列任何情况之一，受试者不应参加试验：

Subjects should not participate in the trial if any of the following conditions occur:

(1) 有严重心血管或循环系统疾病且不能耐受手术；
Have severe cardiovascular or circulatory disease and cannot tolerate surgery;

(2) 妊娠或哺乳；
Pregnancy or lactation;

(3) 有癫痫或精神病史；
History of epilepsy or mental illness;

(4) 严重过敏体质和疑似或已确诊的酒精或药物成瘾者；
Severe allergic constitution and suspected or confirmed alcohol or drug addiction;

(5) 无法理解研究要求，或不能完成研究随访计划；
Inability to understand the study requirements or to complete the study follow-up plan;

(6) 研究者认为不适宜参加本试验。
The investigator did not consider it appropriate to participate in this trial.

3. 停止试验的标准和程序
Criteria and Procedures for Stopping the Test

停止试验是指临床试验尚未按方案结束，中途停止全部临床试验。停止临床试验的目的主要是为了保护受试者权益，保证临床试验质量。除下述的第 5 条外，应由申办者和研究者共同协商是否停止试验：

Discontinuation of the trial means that the clinical trial has not been completed according to the protocol and all clinical trials have been stopped halfway. The main purpose of stopping clinical trials is to protect the rights and interests of subjects and ensure the quality of clinical trials. With the exception of paragraph 5 below, discontinuation of the trial shall be mutually agreed between the sponsor and the investigator:

1) 临床试验过程中发生严重的安全性问题，应及时停止临床试验；
If serious safety problems occur during the clinical trial, the clinical trial should be stopped in time.

2) 临床试验过程中发现产品不具有临床价值，应停止临床试验；
If the product is found to have no clinical value during the clinical trial, the clinical trial shall be stopped;

3) 在临床试验过程中发现临床试验方案有重大失误，难以评价产品效果，或者方案在实施中发生了严重偏差，再继续下去，难以评价产品效果时应停止临床试验；
In the process of clinical trial, it is found that there is a major error in the clinical trial scheme, and it is difficult to evaluate the product effect, or there is a serious deviation in the implementation of the scheme, and then the clinical trial should be stopped when it is difficult to evaluate the product effect.

4) 申办者要求停止（如财务原因等）；
The sponsor requests to stop (such as financial reasons);

5) 国家药品监督管理局因某种原因勒令停止临床试验。
The National Medical Products Administration ordered the clinical trial to be stopped for some reason.

试验停止后，研究者应该从保障受试者权益的角度，继续为未出组的受试者提供适当治疗，并详细告知受试者在其试验期间所接受的治疗及相关处理措施。受试者的相关数据依旧可以用于评价产品的安全性。

After the termination of the trial, the investigator should continue to provide appropriate treatment for the subjects who are not out of the group from the perspective of protecting the rights and interests of the subjects, and inform the subjects in detail of the treatment and related treatment measures they received during the trial. Relevant data from the subjects can still be used to evaluate the safety of the product.

研究者应将受试者试验停止的情况及时反馈给申办者。
The investigator shall give timely feedback to the sponsor on the discontinuation of the subject's trial.

（三）评价方法
(III) Evaluation Method

1. 主要评价指标
Main Evaluation Indicators

（1） 主要有效性评价指标：手术成功率
Main effectiveness evaluation index: success rate of operation

使用试验器械成功完成远程手术治疗的受试者人数，占总受试者人数的比例。手术成功的定义：未从胸腹腔内窥镜手术系统远程手术转换为腹腔镜下手术或者
The number of subjects who successfully completed remote surgical treatment using the test device, as a percentage of the total number of subjects. The definition of successful operation is that there is no conversion from the remote operation of the thoracoabdominal endoscopic surgery system to the laparoscopic operation or

开放性手术。
Open surgery.

（2） 主要安全性评价指标：3 级或以上并发症发生率
Main safety evaluation index: Incidence rate of grade 3 or above complications

从第一个切口到术后 30 天符合 Clavien-Dindo 分级系统 3 级或以上标准的与器械
Clavien-Dindo Grading System Grade 3 or higher from the first incision to 30 days after surgery

肯定有关的或可能有关的并发症发生率。
The incidence of complications that are definitely or possibly related.

2. 次要评价指标
Secondary Evaluation Index

1)整体并发症发生率
Overall complication rate

第一个切口到术后 30 天符合Clavien-Dindo 分级系统 1 级或以上标准的整体并发症发生率。
Overall complication rate from the first incision to 30 days after surgery meeting Clavien-Dindo grading system criteria of grade 1 or higher.

2)手术时间（min）
Operation time (min)

从手术开始到切口缝合结束的总时间。开始切开皮肤的时间为手术开始时间。计算公式：手术时间=手术结束时间-手术开始时间
Total time from the beginning of the procedure to the end of the closure of the incision. The time when the incision of the skin is started is the start time of the operation. Calculation formula: operation time = operation end time-operation start time

3)医生控制台操作时间（min）
Doctor console operation time (min)

从主刀开始操作医生控制台至操作完成离开医生控制台的时间。
The time from the time when the main knife starts to operate the doctor's console to the time when the operation is completed and the doctor's console is left.

4)术中估计失血量（mL）
Estimated intraoperative blood loss (mL)

从手术开始到切口缝合结束的估计失血量。由研究者术后即刻评估。
Estimated blood loss from the start of surgery to the end of incision closure. Evaluated by the investigator immediately after surgery.

5)切缘阳性率 [适用于恶性肿瘤]
Positive margin rate [for malignant tumors]

使用试验器械完成手术，所切除病灶组织切缘阳性的受试者人数，占术后病理诊断为恶性肿瘤受试者人数的比例。
The number of subjects who completed the operation using the test instrument and whose resection margin of the resected lesion tissue was positive accounted for the proportion of the number of subjects who were pathologically diagnosed as malignant tumors after the operation.

6)患者疼痛评分 [术后第 1 天]
Patient pain score [postoperative day 1]

术后采用视觉模拟评分法(VAS)（附录 2）评定术后疼痛，0 分(无疼痛)至 10 分(最大疼痛)。
Postoperative pain was assessed by visual analogue scale (VAS) (Appendix 2), ranging from 0 (no pain) to 10 (maximum pain).

7)医生满意度
Doctor satisfaction

手术后，采用 NASA-TLX 量化表（附录 3）评定医生满意度。 8)术后住院时间（天）
After surgery, physician satisfaction was assessed using the NASA-TLX Quantification Form (Appendix 3). 8) Postoperative hospital stay (days)

手术当天至出院当天的住院治疗天数。
Days of hospitalization from the day of surgery to the day of discharge.

计算公式：术后住院天数=出院当天日期-手术当天日期
Calculation formula: postoperative hospital stay = date of discharge-date of operation

9)输血率
Blood transfusion rate

受试者手术开始至术后 30 天内，发生输注红细胞的人数占总人数的比例。
The proportion of the number of subjects with red blood cell transfusion from the beginning of the operation to 30 days after the operation.

10)再入院率
Readmission rate

受试者手术后恢复出院，术后 30 天内因手术相关且器械相关原因再次住院治疗
The subject was discharged from hospital after recovery from surgery and was readmitted within 30 days of surgery for both procedure-related and device-related reasons.

的人数占总人数的比例。
The proportion of the population to the total population.

11) 再次手术率
Reoperation rate

受试者手术结束并离开手术室后的 30 天内，因手术相关且器械相关原因再次进入手术室进行手术治疗的人数占总人数的比例。
The percentage of subjects who were re-admitted to the operating room for surgery-related and device-related reasons within 30 days after the end of surgery and leaving the operating room.

12) 死亡率
Mortality

受试者术后 30 天内发生的与手术相关且器械相关的死亡人数占总人数的比例。
The proportion of procedure-related and device-related deaths that occurred within 30 days of the subject's surgery.

13) 器械缺陷发生率
Incidence of device defects

手术过程中，发生系统故障、器械失效、器械损坏导致手术无法完成的人数占总人数的比例。
During the operation, the proportion of the total number of people who can not complete the operation due to system failure, instrument failure and instrument damage.

14) 不良事件发生率
Incidence of adverse events

受试者手术开始至术后 30 天内，发生器械相关的不良事件的人数占总人数的比例。
The proportion of the number of subjects with device-related adverse events from the start of surgery to 30 days after surgery to the total number of subjects.

15) 严重不良事件发生率
Incidence of serious adverse events

受试者手术开始至术后 30 天内，发生器械相关的严重不良事件的人数占总人数的比例。
The proportion of the number of subjects with device-related serious adverse events from the start of surgery to 30 days after surgery to the total number of subjects.

16) 远程操作评价
Remote operation evaluation

研究者填写远程手术操作评分表（附录 4）和远程手术网络传输记录表（附录 5），评定远程指标。
The researchers filled in the remote surgical operation scoring table (Appendix 4) and the remote surgical network transmission record table (Appendix 5) to evaluate the remote index.

（3） 确定依据
Basis of determination

根据《腹腔内窥镜手术系统技术审评要点》（2023 年修订版），确定本试验的主、次要有效性评价指标。
The primary and secondary effectiveness evaluation indexes of this trial were determined according to the Key Points for Technical Review of Laparoscopic Surgery System (revised edition in 2023).

3. 整机安全性评价和不良事件、严重不良事件
Safety Evaluation of the Whole Machine, Adverse Events and Serious Adverse Events

（1） 整机安全性评价
Safety evaluation of the whole machine

以下情况被定义为系统故障，作为整机安全性评价的依据：
The following conditions are defined as system faults, which are used as the basis for safety evaluation of the whole machine:

①系统运行出错，无法通过用户操作界面进行恢复且发生与对应器械相关的不良事件。
① The system runs in error and cannot be restored through the user operation interface, and adverse events related to the corresponding device occur.

②手术器械夹持时不能松开组织且无法通过夹钳紧急释放装置松开。
② The surgical instrument cannot release the tissue during clamping and cannot be released by the clamp emergency release device.

③手术过程中手术器械的失效或损坏且发生与对应器械相关的不良事件。
③ Failure or damage of surgical instruments and occurrence of adverse events related to corresponding instruments during the operation.

④手术过程中发生网络中断、网络传输延迟、带宽波动、抖动、丢帧且发生与对应器械相关的不良事件。

④ Network interruption, network transmission delay, bandwidth fluctuation, jitter, frame loss and adverse events related to corresponding devices occurred during the operation.

以上指标为定性指标，由操作者进行判断，判断结果为“有”或“无”。

The above indicators are qualitative indicators, which are judged by the operator, and the judgment result is "yes" or "no".

注：在维修前同一原因连续发生的故障，属一次故障。如故障发生后得到修正，在24小时内采用相同的流程未复现同样的故障现象，则认为本故障已解决。

Note: The continuous failure caused by the same reason before maintenance belongs to one failure. If the fault is corrected and the same fault phenomenon is not reproduced within 24 hours by using the same process, the fault is considered to have been solved.

器械失效的定义：指器械正确安装后不能实现主要功能。

Definition of device failure: means that the main function of the device cannot be realized after the device is correctly installed.

器械损坏的定义：指正常使用过程中，发生结构部件或外观破坏。

Definition of device damage: refers to the damage of structural components or appearance during normal use.

（2） 记录不良事件和严重不良事件

Record adverse events and serious adverse events

对于试验过程中发生的不良事件和严重不良事件，需记录于电子病例报告表中，并判定不良事件和严重不良事件是否与受试器械有关。不良事件与受试器械的关系，分为“肯定无关”、“可能无关”、“可能有关”、“肯定有关”，通过不良事件、严重不良事件发生率和与器械相关的不良事件、严重不良事件反映受试器械的安全性。

Adverse events and serious adverse events occurred during the trial shall be recorded in the electronic case report form, and whether the adverse events and serious adverse events are related to the test device shall be determined. The relationship between adverse events and the device under test is divided into "definitely unrelated", "possibly unrelated", "possibly related" and "definitely related". The safety of the device under test is reflected by the incidence of adverse events, serious adverse events and adverse events and serious adverse events related to the device.

（3） 评价、记录和分析安全性参数的方法和时间选择

Method and timing of evaluation, recording and analysis of safety parameters

临床试验过程中，记录系统故障发生次数以及发生的不良事件/严重不良事件，验证产品的安全性。

During the clinical trial, record the number of system failures and adverse events/serious adverse events to verify the safety of the product.

（4） 确定依据

Basis of determination

根据试验器械的适用范围、产品特点、工作原理确定本试验的主、次要安全性评价指标。

The primary and secondary safety evaluation indexes of the test are determined according to the scope of application, product characteristics and working principle of the test device.

（四）试验医疗器械和对照医疗器械

(IV) Test Medical Devices and Control Medical Devices

1. 试验医疗器械

Test Medical Device

名称：胸腹腔内窥镜手术系统

Name: Thoracic and Abdominal Endoscopic Surgery System

型号：MP1000

Model: MP1000

生产企业：深圳市精锋医疗科技股份有限公司

Manufacturer: Shenzhen Jingfeng Medical Technology Co., Ltd.

适用范围：该产品由医师利用主从操控系统对于微创手术器械进行控制，用于泌尿外科、妇科、普通外科和胸外科手术。荧光成像功能用于提供手术部位的可见光及近红外光成像，其中近红外光成像需配合已在中国批准上市且应用部位一致的吲哚菁绿使用。远程功能用于提供远程手术操作。

Scope of application: The product is controlled by the master-slave control system for minimally invasive surgical instruments, and is used in urology, gynecology, general surgery and thoracic surgery. The fluorescence imaging function is used to provide visible light and near-infrared light imaging of the surgical site, in which the near-infrared light imaging needs to be used in conjunction with indocyanine green, which has been approved for marketing in China and has the same application site. The remote function is used to provide remote surgical operation.

2. 对照医疗器械

Control Medical Device

本研究为前瞻性、多中心、单组临床试验，不涉及对照医疗器械。

This study was a prospective, multicenter, single-arm clinical trial and did not involve a controlled medical device.

（五）试验流程

(V) Test Process

1. 试验流程表

Test Flow Chart

阶段<br>Phases	筛选期<br>Screening period	随访<br>Follow-up	
	第 1 次访视<br>Visit 1	第 2 次访视<br>Visit 2	第 3 次访视<br>Visit 3
	术前-10~0D<br>-10 ~ 0 D before operation	术中<br>During the operation<br>或术后即刻+3D<br>Or immediately after surgery + 3D	术后 30+10D<br>Postoperative 30 + 10 D
签署知情同意<br>Sign the informed consent	●		
填写基本资料①<br>Complete Basic Information ①	●		
术前诊断<br>Preoperative diagnosis	●		
采集病史②<br>Take medical history ②	●		
生命体征检查③<br>Vital signs ③	●	●	●
生化检查④<br>Biochemical test ④	●	●	●
血常规检查⑤<br>Blood routine examination ⑤	●	●	●
尿常规检查⑥<br>Routine urinalysis ⑥	●		
血/尿 HCG 检查⑦<br>Blood/Urine HCG Test ⑦	●		
凝血功能检查⑧<br>Coagulation function test ⑧	●		
心电图检查⑨<br>ECG ⑨	●		
手术部位CT 或 MR<br>Surgical site CT or MR	●		
手术部位其他影像学检查<br>Other imaging examinations of the surgical site	●		
筛选/入组<br>Screening/Grouping	●		
手术信息<br>Surgical information		●	

手术时间<br>Time of operation		●	
术中估计失血量<br>Estimated intraoperative blood loss		●	
手术是否成功<br>Whether the operation was successful		●	

术后病理切缘检查⑩<br>Postoperative pathological examination of incisal margin ⑩		●	
整机安全性评价表<br>Safety evaluation table of the whole machine		●	
患者疼痛评分<br>Patient pain score		●	
医生满意度<br>Doctor satisfaction		●	
远程操作评价<br>Remote operation evaluation		●	
器械缺陷<br>Device Defect		●	
并发症<br>Complications		●	●
住院时间<br>Length of stay			●
记录不良事件⑪<br>Record adverse events ⑪		●	●
合并用药⑫<br>Concomitant Medications ⑫	●	●	●
是否输血<br>Blood transfusion or not		●	●
是否再入院<br>Readmission or not			●
是否再次手术<br>Whether to operate again		●	●

备注：
Remar:

① 患者基本资料包括出生年月、性别、体重、身高、民族。
① The basic information of the patients included birth date, sex, weight, height and nationality.

② 病史采集：病史包括既往手术史、疾病史（包括既往确诊的疾病和筛选期发现的疾病）。
② Medical history collection: Medical history includes previous surgical history and disease history (including previously diagnosed diseases and diseases found in the screening period).

③ 生命体征包括脉率、呼吸频率、收缩压和舒张压、体温。
③ Vital signs include pulse rate, respiratory rate, systolic and diastolic blood pressure, and temperature.

④ 生化检查：记录丙氨酸氨基转移酶、天冬氨酸氨基转移酶、总蛋白、白蛋白、肌酐、尿酸、钾。若受试者签署知情同意书前 10 天内进行过生化检查、血常规检查、尿常规检查、血/尿 HCG 检查、凝血功能检查，接受检查结果作为本试验筛选期数据，且可接受外院检查结果。

④ Biochemical examination: record alanine aminotransferase, aspartate aminotransferase, total protein, albumin, creatinine, uric acid and potassium. If the subject has undergone biochemical examination, blood routine examination, urine routine examination, blood/urine HCG examination and coagulation function examination within 10 days before signing the informed consent, the examination results shall be accepted as the data of the screening period of this trial, and the examination results of other hospitals can be accepted.

⑤ 血常规：血红蛋白浓度、红细胞计数、白细胞计数、中性粒细胞比值和血小板。

⑤ Blood routine examination: hemoglobin concentration, red blood cell count, white blood cell count, neutrophil ratio and platelet count.

⑥ 尿常规: 尿白细胞、尿红细胞和尿蛋白。

⑥ Urine routine: white blood cells, red blood cells and protein in urine.

⑦ 血/尿 HCG 检查：所有育龄期女性受试者都进行 HCG 检查，除非病史中记录有受试者做过绝育手术（子宫切除术、卵巢切除术或两侧输卵管结扎）或绝经时间至少 2 年。

⑦ Blood/urine HCG examination: HCG examination was performed on all female subjects of childbearing age, unless it was documented in the medical history that the subject had undergone sterilization (hysterectomy, oophorectomy, or bilateral tubal ligation) or had been menopausal for at least 2 years.

⑧ 凝血功能检查：PT、APTT、INR。

⑧ Coagulation function test: PT, APTT, INR.

⑨ 心电图检查：若受试者签署知情同意书前 30 天内进行过心电图和本方案要求进行的影像学检查，接受检查结果作为本试验筛选期数据，接受外院检查结果。

⑨ Electrocardiogram examination: If the subject has undergone electrocardiogram and imaging examination required by this protocol within 30 days before signing the informed consent, the examination results shall be accepted as the data of the screening period of this trial, and the examination results of other hospitals shall be accepted.

⑩ 术后病理切缘检查：恶性肿瘤患者术后病理注意需标明切缘是否阳性。

⑩ Postoperative pathological examination of resection margin: Patients with malignant tumors should pay attention to whether the resection margin is positive or not.

⑪ 记录不良事件：从受试者手术开始监测不良事件，术后常见应激反应不作为不良事件记录，如切口疼痛、胃肠功能紊乱、皮肤麻木、留置尿导管导致的尿路刺激等症状，如果事件导致 SAE 则需记录。

⑪ Recording of adverse events: adverse events were monitored from the beginning of the subject's operation. Common stress reactions after operation were not recorded as adverse events, such as incision pain, gastrointestinal dysfunction, skin numbness, urinary tract irritation caused by indwelling urinary catheter, and other symptoms. If the event resulted in SAE, it should be recorded.

⑫ 合并用药：签署知情同意书后开始记录合并用药，包括当天仍在使用的药物。

⑫ Concomitant medications: After signing the informed consent form, start recording the concomitant medications, including the medications still in use on that day.

2. 试验实施（方法、内容、步骤等）
Test Implementation (methods, Contents, Steps, Etc.)

在治疗过程中，所有与临床常规治疗相关检查均应按照临床需要，在医生指导下进行。研究流程设计如下：

In the course of treatment, all examinations related to clinical routine treatment should be carried out under the guidance of doctors according to clinical needs. The research process is designed as follows:

⑴ 第一次访视：研究者充分告知受试者临床试验详细情况，双方签署《知情同意书》，分配筛选号，填写受试者筛选/入选表、受试者鉴认代码表，记录受试者基本信息（年龄、性别、诊断结果等），记录患者既往病史，安排受试者进行生命体征检查、筛选期实验室检查（包括血常规、尿常规、血/尿 HCG、生化检查、凝血功能等）、心电图检查、手术部位 CT 或 MR 等相关影像学检查。记录筛选期合并用药。

The first visit: the investigator fully informed the subject of the details of the clinical trial, both parties signed the Informed Consent, assigned the screening number, filled in the subject screening/selection form and the subject identification code form, recorded the basic information of the subject (age, gender, diagnosis results, etc.), and recorded the past medical history of the patient. Vital signs examination, laboratory examination (including blood routine, urine routine, blood/urine HCG, biochemical examination, coagulation function, etc.), electrocardiogram examination, CT or MR of the surgical site and other related imaging examinations were arranged for the subjects during the screening period. Concomitant medications during the screening period were recorded.

⑵ 第二次访视：远程端和患者端进行术前准备，远程端启动医生控制台，患者端启动影像系统和患者手术平台。工程师团队进行医生控制台与患者手术平台之间的网络连接和设备调试。调试合格后，将受试者麻醉，将患者手术平台置于适当位置，根据手术要求放置机械臂，将机械臂和套管进行对接，安装手术器械。远程端主刀医生通过医生控制台控制患者端的患者手术平台，控制机械臂开始进行手术操作。

The second visit: the remote end and the patient end carry out preoperative preparation,

the remote end starts the doctor console, and the patient end starts the imaging system and the patient operation platform. The team of engineers performs the network connection and equipment commissioning between the doctor's console and the patient's surgical platform. After the debugging is qualified, the subject is anesthetized, the patient's surgical platform is placed in an appropriate position, the mechanical arm is placed according to the surgical requirements, the mechanical arm and the cannula are docked, and the surgical instruments are installed. The chief surgeon at the remote end controls the patient operation platform at the patient end through the doctor console, and controls the mechanical arm to start the operation.

手术结束后，记录手术时间、术中估计失血量、手术是否成功、合并用药、器械缺陷发生情况以及受试者生命体征，受试者手术后进行血常规、血生化以及病理切片检查，研究者填写 NASA-TLX 量化表、远程手术操作评分表、远程手术网络传输记录表。受试者采用视觉模拟评分法（VAS）进行疼痛评分，记录并发症、不良事件、合并用药、是否发生输血及是否再次手术。

After the operation, the operation time, the estimated blood loss during the operation, the success of the operation, the concomitant medication, the occurrence of instrument defects and the vital signs of the subjects were recorded. After the operation, the subjects were examined by blood routine, blood biochemistry and pathological section. The investigators filled in the NASA-TLX quantification table, the remote operation score table and the remote operation network transmission record table. Visual analogue scale (VAS) was used to score pain, and complications, adverse events, concomitant medication, blood transfusion and reoperation were recorded.

⑶ 第三次访视：受试者进行生命体征、血常规、生化检查，记录并发症、住院时间、不良事件、合并用药，并记录是否发生输血、是否发生再入院、是否发生再次手术。在完成第 3 次访视后出组。

The third visit: the subjects were given vital signs, blood routine and biochemical examination, and the complications, length of stay, adverse events, combined medication, blood transfusion, readmission and reoperation were recorded. Leave the group after the completion of visit 3.

⑷ 由统计人员分析，各临床试验机构出具小结报告，牵头单位组织汇总形成临床试验报告。

Statisticians analyze, clinical trial institutions issue summary reports, and leading units organize and summarize to form clinical trial reports.

3. 用械规范

Equipment Specification

(1) 申办者应当免费提供试验医疗器械，并符合以下要求：
(1) The sponsor shall provide the trial medical device free of charge and meet the following requirements:

1) 试验医疗器械应当按照医疗器械生产质量管理规范的相关要求生产且质量合格；

The trial medical devices shall be manufactured in accordance with the relevant requirements of the Good Manufacturing Practice for Medical Devices and shall be qualified;

2) 确定试验医疗器械的运输条件、储存条件、有效期等；
Determine the transportation conditions, storage conditions, validity period, etc. of the test medical device;

3) 试验医疗器械应当按照临床试验方案要求进行适当包装和保存；包装标签上应当标明产品信息，具有易于识别、正确编码的标识，标明仅用于医疗器械临床试验；

The trial medical devices shall be properly packaged and preserved in accordance with the requirements of the clinical trial scheme; The product information shall be marked on the package label, which shall be easily identified and correctly coded, and shall be used only for clinical trials of medical devices;

4) 医疗器械临床试验获得伦理委员会同意后，申办者负责在规定的条件下将试验医疗器械运输至医疗器械临床试验机构；

After the medical device clinical trial is approved by the ethics committee, the sponsor is responsible for transporting the trial medical device to the medical device clinical trial institution under the specified conditions;

5) 对从医疗器械临床试验机构回收的试验医疗器械，申办者负责保存回收处置等记录。

For the trial medical devices recovered from the medical device clinical trial institution, the sponsor shall be responsible for keeping the records of recovery and disposal.

(2) 研究者：
(2) Investigator:

1) 研究者对申办者提供的试验医疗器械有管理责任，应当确保其仅用于参加该医疗器械临床试验的受试者，在临床试验期间按照要求储存和保管，在临床试验完成或者终止后按照相关法律法规和与申办者的合同进行处理。

The investigator shall be responsible for the management of the trial medical device provided by the sponsor, and shall ensure that it is only used for the subjects participating in the clinical trial of the medical device, stored and kept as required during the clinical trial, and handled in accordance with relevant laws and regulations and the contract with the sponsor after the completion or termination of the clinical trial.

2) 参与本临床试验的研究者均具有腔镜手术相关工作经验，保证操作的熟练性

和规范性。

The investigators participating in this clinical trial all have relevant working experience in endoscopic surgery to ensure the proficiency and standardization of the operation.

4. 合并用药规范
Code of Practice for Concomitant Medication

1) 签署知情同意书后开始记录合并用药。
Concomitant medications were recorded after informed consent was signed.

2) 皮试用药、配液、营养补液、麻醉相关药物、术后常规及预防用药不作为合并用药记录。

Skin test medication, fluid preparation, nutritional rehydration, anesthesia-related medication, postoperative routine and preventive medication were not recorded as combined medication.

3) 记录药物通用名、用药原因、剂量、剂量单位、给药频率、给药途径、开始用药时间、结束用药时间、是否持续。

Record the generic name of the drug, the reason for administration, the dosage, the dosage unit, the frequency of administration, the route of administration, the time when the drug was started, the time when the drug was stopped, and whether it was continued.

（六）偏倚控制措施
(VI) Bias Control Measures

1.临床试验开始前，申办者对参与研究的研究者进行相关培训，确保研究者充分了解研究流程，熟练操作试验器械，严格按照方案进行测试，避免研究过程中因违反方案而产生的偏差。

Before the start of the clinical trial, the sponsor shall provide relevant training for the investigators participating in the study to ensure that the investigators fully understand the study process, skillfully operate the test instruments, and conduct the test in strict accordance with the protocol, so as to avoid deviations caused by violation of the protocol during the study.

2.严格根据试验方案的入选和排除标准对受试者进行筛选，减少选择性偏倚。
Subjects were screened strictly according to the inclusion and exclusion criteria of the trial protocol to reduce selection bias.

3.临床试验过程中，研究者必须严格按照临床方案中的操作方法及规程进行操作，临床试验监查员应做好质量控制与监查工作，确保研究者严格按照临床方案进行操作、实施。以上措施贯彻在整个临床的实施阶段，以减少过失或操作误差。

During the clinical trial, the investigator must operate in strict accordance with the operating methods and procedures in the clinical protocol, and the clinical trial supervisor shall do a good job in quality control and supervision to ensure that the investigator operates and implements in strict accordance with the clinical protocol. The above measures are carried out throughout the clinical implementation phase to reduce errors or operational errors.

4.参与本临床试验的研究者均具有腔镜手术相关工作经验，保证了操作的熟练性和规范性。

The investigators participating in this clinical trial all have relevant experience in endoscopic surgery, which ensures the proficiency and standardization of the operation.

5.临床试验方案建立了统一的记录方法和判断标准，避免了研究者主观倾向造成的偏倚。

The clinical trial protocol has established a unified recording method and judgment criteria, which avoids the bias caused by the subjective tendency of researchers.

6.临床研究完成时，使用电子数据采集系统（EDC）对试验数据进行录入和验证，当发现数据问题时，监查员、数据管理员等在 EDC 中发出人工疑问，研究者需及时解答疑问。数据管理员和监查员进行疑问批复，必要时可再次发出疑问，直至数据“清洁”。

When the clinical study is completed, the electronic data acquisition system (EDC) is used to input and verify the test data. When data problems are found, the monitors and data managers send out manual questions in the EDC, and the researchers need to answer the questions in time. The data administrator and the monitor shall reply to the query, and may issue the query again if necessary, until the data is "clean".

六、统计学考虑
VI. Statistical Considerations

（一）样本量估算
(I) Sample Size Estimation

1. 样本量
Sample Size

根据《腹腔内窥镜手术系统技术审评要点（2023 年修订版）》 中临床试验总体设计要求，经临床试验机构、主要研究者、统计分析师和申办者共同研究讨论，计划入组 6 例。

According to the overall design requirements of the clinical trial in the Key

Points for Technical Review of Laparoscopic Surgery System (Revised in 2023), 6 cases were planned to be enrolled after the joint study and discussion of the clinical trial institution, the principal investigator, the statistical analyst and the sponsor.

2. 样本量分配以及其确定依据

Sample size allocation and the basis for its determination

本次临床试验计划纳入的手术主要为肝脏部位的典型代表术式。每家中心根据预期病源数进行病例入组，试验结束后对此信息进行汇总。

The operations included in this clinical trial plan are mainly the typical representative operations in the liver. Each center enrolled cases according to the expected number of disease sources, and summarized this information after the end of the trial.

（二）分析数据集

(II) Analytical Data Set

1.全分析集（FAS）：指合格病例和脱落病例的集合，但不包含剔除病例。尽可能根据意向性分析（intention to treat，ITT分析）确定的数据集：指所有使用研究器械的受试者所构成的数据集。

Full analysis set (FAS): refers to the set of qualified cases and dropped cases, but does not include the rejected cases. Data set determined by intention to treat (ITT analysis) as far as possible: refers to the data set composed of all subjects using the study device.

2.符合方案集（PPS）：指符合试验方案规定的入选标准、不符合排除标准、完成全部计划访视的病例，即对符合试验方案、依从性好、完成CRF规定填写内容的病例进行分析（PP分析）。其是FAS的一个子集，在这个数据集中每位受试者依从性好，

Per protocol set (PPS): refers to the cases that meet the inclusion criteria specified in the trial protocol, do not meet the exclusion criteria, and complete all planned visits, that is, the cases that meet the trial protocol, have good compliance, and complete the contents specified in the CRF are analyzed (PP analysis). It is a subset of FAS, in which each subject has good compliance.

不违背方案，主要指标的基线值完备。可以从 FAS 中排除的情况：1)主要指标无基线值；2)严重违背方案；3)依从性差。

The scheme is not violated, and the baseline values of the main indicators are complete. Conditions that can be excluded from the FAS: 1) no baseline value for the primary indicator; 2) serious protocol violation; 3) Poor compliance.

3.安全分析集（SS）：所有入组病例中至少使用研究器械且有安全性评价数据的病例。安全性缺失值不得结转。

Safety analysis set (SS): All enrolled cases at least used the study device and had safety evaluation data. Security missing values shall not be carried forward.

（三）受试者剔除标准
(III) Subject Exclusion Criteria

统计分析前，由主要研究者、申报者、统计分析人员判断个例是否剔除。出现下列情况之一，三方人员将依据受试者完成试验的程度和退出原因等因素综合判断是否将此受试者剔除，并作出相关说明。

Before the statistical analysis, the main researcher, the applicant and the statistical analyst shall judge whether the case is excluded. In case of any of the following circumstances, the personnel of the three parties will comprehensively judge whether to exclude the subject according to the degree of completion of the test and the reasons for withdrawal, and make relevant explanations.

1. 受试者个例的选择不符合入选标准或符合排除标准，且影响主要评价指标者；
The selection of individual subjects did not meet the inclusion criteria or met the exclusion criteria, and affected the main evaluation indicators;

2. 入组后未使用试验器械，无任何观察数据；或者记录不完整，无法作出有效性评价。

The test instrument was not used after enrollment, and there was no observation data; Or the records are incomplete and the effectiveness evaluation cannot be made.

（四）统计方法
(4) Statistical Methods

1. 统计分析原则
Principles of Statistical Analysis

统计分析所采用的软件为 SAS9.4 或以上版本统计软件，试验结果统计分析有效性指标同时对全分析数据集和符合方案数据集进行分析，安全性指标对安全性数据集进行分析。

The software used for statistical analysis is SAS 9.4 or above version statistical software. The validity index of statistical analysis of test results analyzes the full analysis data set and the data set in accordance with the scheme, and the safety index

analyzes the safety data set.

定量指标的描述将计算均值、标准差、中位数、最小值、最大值，下四分位数（Q1），上四分位数（Q3），分类指标描述各类的例数及百分数。

The description of quantitative indicators will calculate the mean, standard deviation, median, minimum, maximum, lower quartile (Q1), upper quartile (Q3), and the number of cases and percentage of each category described by the classification indicators.

2. 完成情况及人口学分析
Completion and Demographic Analysis

基线分析基于全数据集（FAS）。
The baseline analysis was based on the full data set (FAS).

总结各中心入组及完成数，列出脱落病例的清单。数据集大小、各中心病例分布、总脱落率比较、未完成原因详细列表。对患者的人口学特征(年龄、身高、生命体征等)、病史、用药史及可配器械的使用情况等进行描述。

The enrollment and completion number of each center were summarized, and the list of dropped cases was made. Data set size, distribution of cases by center, comparison of total drop-out rates, detailed list of reasons for non-completion. Describe the patient's demographic characteristics (age, height, vital signs, etc.), medical history, medication history, and the use of available devices.

3. 主要评价指标
Main Evaluation Indicators

1) 主要有效性评价指标
Main effectiveness evaluation index

主要有效性评价基于全数据集（FAS）和符合方案集（PPS）。数据的统计描述与推断将根据数据的特征，选择适用的描述指标和统计方法。

The primary effectiveness evaluation was based on the full data set (FAS) and the per-protocol set (PPS). The statistical description and inference of data will select the applicable descriptive indicators and statistical methods according to the characteristics of the data.

2) 主要安全性评价指标
Main safety evaluation indexes

主要安全性评价基于全数据集（FAS）和符合方案集（PPS）。数据的统计描述与推断将根据数据的特征，选择适用的描述指标和统计方法。

The primary safety assessment was based on the full data set (FAS) and the per-protocol set (PPS). The statistical description and inference of data will select the applicable descriptive indicators and statistical methods according to the characteristics of the data.

4.次要评价指标
Secondary Evaluation Index

1）主要有效性评价指标
Main effectiveness evaluation index

次要有效性评价基于全数据集（FAS）和符合方案集（PPS）。数据的统计描述与推断将根据数据的特征，选择适用的描述指标和统计方法次要有效性评价指标包括：手术时间、医生控制台操作时间、切缘阳性率、患者疼痛评分、医生满意度、术后住院时间、再入院率、再次手术率、远程操作评价。

Secondary effectiveness evaluations were based on the full data set (FAS) and the per-protocol set (PPS). The statistical description and inference of the data will be based on the characteristics of the data, and the applicable descriptive indicators and statistical methods will be selected. The secondary effectiveness evaluation indicators include: operation time, doctor console operation time, positive margin rate, patient pain score, doctor satisfaction, postoperative hospital stay, readmission rate, reoperation rate, and remote operation evaluation.

2）次要安全性评价指标
Secondary safety evaluation index

次要安全性评价在 SS 数据集上进行分析。数据的统计描述与推断将根据数据的特征，选择适用的描述指标和统计方法。次要有效性评价指标包括：整体并发症发生率、术中估计失血量、输血率、死亡率、器械缺陷发生率、不良事件发生率、严重不良事件发生率。

Secondary safety assessments were analyzed on the SS data set. The statistical description and inference of data will select the applicable descriptive indicators and statistical methods according to the characteristics of the data. Secondary efficacy measures included overall complication rate, estimated intraoperative blood loss, blood transfusion rate, mortality rate, device defect rate, adverse event rate, and serious adverse event rate.

5. 整机安全性评价和不良事件、严重不良事件
Safety Evaluation of the Whole Machine, Adverse Events and Serious Adverse Events

列表描述试验产品系统故障发生的例数，不良事件和与器械相关的不良事件的例

次和人数，以及严重不良事件和与器械相关的严重不良事件的例次和人数，把不良事件和与器械相关的不良事件、严重不良事件和与器械相关的严重不良事件列清单描述。

List the number of cases of system failure of the test product, the number and number of cases of adverse events and adverse events related to the device, as well as the number and number of cases of serious adverse events and serious adverse events related to the device, and list the adverse events and adverse events related to the device, serious adverse events and serious adverse events related to the device.

（五）缺失值和异常值的处理
(V) Handling of Missing Values and Abnormal Values

1. 缺失值处理
Missing Value Processing

对缺失数据，FAS 集采用最差值进行填补；其他指标的缺失值不做估计。
For the missing data, the FAS set is filled with the worst value. Missing values for other indicators are not estimated.

2. 不合理数据处理
Unreasonable Data Processing

数据管理过程中，将对数据库中数据进行逻辑性核查，发现不合理数据，监查员、数据管理员等在 EDC 中发出人工疑问，研究者需及时解答疑问。数据管理员和监查员进行疑问批复，必要时可再次发出疑问，直至所有不合理数据获得解决，方可锁定数据库。

In the process of data management, the data in the database will be checked logically. If unreasonable data is found, the inspector and data administrator will send out artificial questions in EDC, and the researcher needs to answer the questions in time. The data administrator and the monitor shall reply to the query, and may issue the query again if necessary, until all unreasonable data are resolved, and then the database can be locked.

3. 错误数据处理
Error Data Processing

数据管理过程中，将对数据库中数据进行质量管理，发现错误数据，监查员、数据管理员等在 EDC 中发出人工疑问，研究者需及时解答疑问。数据管理员和监查员进行疑问批复，必要时可再次发出疑问，直至所有错误数据获得解决，方可锁定数据库。

In the process of data management, the quality of data in the database will be managed. If wrong data is found, the inspector and data administrator will send out artificial questions in EDC, and the researcher needs to answer the questions in time. The data administrator and the monitor shall reply to the query and issue the query again if necessary. The database can not be locked until all the wrong data are solved.

七、监查计划

VII. Audit Plan

1. 由申办者委托的监查员负责对本临床试验进行监查。
   The monitor entrusted by the sponsor is responsible for monitoring the clinical trial.

2. 监查员应有相应的临床医学、药学、生物医学工程、统计学等相关专业背景，并经过必要的培训，熟悉医疗器械临床试验管理规范和有关法规，熟悉有关试验用医疗器械的非临床和同类产品临床方面的信息以及临床试验方案及其相关的文件。

The monitors shall have corresponding professional background in clinical medicine, pharmacy, biomedical engineering, statistics and other related disciplines, and shall receive necessary training, be familiar with the management specifications and relevant laws and regulations for clinical trials of medical devices, and be familiar with the clinical information of non-clinical and similar products of medical devices used in the trial, as well as the clinical trial scheme and its related documents.

3. 根据入组情况，适时进行监查。
   According to the enrollment situation, timely supervision should be carried out.

4. 监查员应遵循 GCP 原则，督促临床试验的进行，以保证临床试验按方案严格执行。试验数据真实、完整、准确。具体职责包括：

The monitor shall follow the GCP principle and supervise the clinical trial to ensure that the clinical trial is carried out in strict accordance with the protocol. The test data is true, complete and accurate. Specific responsibilities include:

⑴ 在试验前确认临床试验机构已具有适当的条件，包括人员配备与培训，实验室设备齐全，工作情况良好，估计有足够数量的受试者，参与研究人员熟悉试验要求；

Before the trial, confirm that the clinical trial institution has appropriate conditions, including staffing and training, complete laboratory equipment, good working conditions, estimated sufficient number of subjects, and the participants are familiar with the requirements of the trial;

⑵ 在试验前、中、后期监查临床试验机构和研究者是否遵从已批准的临床试验方案、医疗器械临床试验管理规范或有关法规；

Monitor whether clinical trial institutions and investigators comply with the approved clinical trial protocol, medical device clinical trial management specifications or

relevant regulations before, during and after the trial;

⑶ 确认每位受试者在参与试验前签署知情同意书，了解受试者的入选率及试验的进展状况。对研究者未能做到的随访、未进行的试验、未做的检查，以及是否对错误、遗漏做出纠正等，应清楚、如实记录。对修订的知情同意书，确认未结束访视的受试者重新签署；

Confirm that each subject signs the informed consent before participating in the trial, and understand the enrollment rate of subjects and the progress of the trial. The follow-up, test and examination that the investigator failed to do, as well as whether the errors and omissions were corrected, should be clearly and truthfully recorded. For the revised informed consent form, confirm that the subjects who have not finished the visit have signed it again;

⑷ 确认所有病例报告表填写正确，并与原始资料一致。所有错误或遗漏均已改正或注明，经研究者签名并注明日期。试验的病种、病例总数和病例的性别、年龄、治疗效果等均应确认并记录；

Confirm that all case report forms are filled in correctly and are consistent with the original data. All errors or omissions were corrected or noted, signed and dated by the investigator. The disease type, total number of cases, gender, age and treatment effect of the cases shall be confirmed and recorded;

⑸ 确认受试者退出研究或不依从知情同意书规定要求的情况记录在案，并与研究者讨论此种情况；

Document and discuss with the investigator any instances of withdrawal from the study or non-compliance with the requirements of the informed consent;

⑹ 确认所有不良事件和器械缺陷均已记录在案，严重不良事件和可能导致严重不良事件的重大器械缺陷在规定时间内做出报告并记录在案；

Confirm that all adverse events and device defects have been recorded, and serious adverse events and major device defects that may lead to serious adverse events have been reported and recorded within the specified time;

⑺ 负责对试验用医疗器械样品的供给、储藏、使用、维护及试验后医疗器械样

Be responsible for the supply, storage, use and maintenance of medical device samples for the test and the medical device samples after the test

品的处理过程进行监查；
Supervise the treatment process of the product;

⑻ 确保临床试验评估相关设备进行定期维护和校准，并记录在案；
Ensure regular maintenance and calibration of clinical trial evaluation-related equipment is maintained and documented;

⑼ 确保研究者收到临床试验相关的所有文件的最新版文件；
Ensure that the investigator receives the latest version of all documents related to the clinical trial;

⑽ 每次监查后应当做一书面报告递送申办者，报告应述明监查日期、时间、监查员姓名、监查地点、研究者姓名、检查内容、项目完成情况、监查的发现、事实、偏离、结论以及对错误、遗漏做出的纠正等。

After each audit, a written report shall be made and submitted to the sponsor, stating the date and time of the audit, the name of the auditor, the place of the audit, the name of the investigator, the contents of the inspection, the completion of the project, the findings, facts, deviations, conclusions of the audit, and the corrections made to the errors and omissions.

八、数据管理
VIII. Data Management

（一）EDC数据管理
(I) EDC Data Management

本次试验采用电子化数据管理，使用 EDC。以下列出数据管理主要流程，其他详见数据管理计划（DMP）。DMP作为数据管理的指导性文件由数据管理员（DM）撰写，申办方批准，数据管理工作将根据 DMP定义的时间、内容及方法进行。

Electronic data management and EDC were used in this test. The main data management processes are listed below, and others are detailed in the Data Management Plan (DMP). The DMP, as a guidance document for data management, is written by the Data Manager (DM) and approved by the Sponsor. Data management will be carried out according to the time, content and method defined by the DMP.

1.电子病例报告表（eCRF）：数据管理员根据试验方案设计构建，并根据数据核查计划（DVP）设置逻辑核查，通过测试并获申办方批准后发布使用。

Electronic case report form (eCRF): it is designed and constructed by the data administrator according to the trial protocol, set up logical check according to the data check plan (DVP), and released for use after passing the test and being approved by the sponsor.

2.数据录入：eCRF数据来源于原始记录，由数据录入人员根据 eCRF填写说明，将受试者访视数据及时录入 EDC。

Data entry: eCRF data comes from the original records, and the data entry personnel

shall enter the subject visit data into the EDC in time according to the eCRF filling instructions.

3. 源数据现场核查（SDV）：监查员进行 eCRF 数据与源数据的一致性核对，有问题可发疑问。

On-site verification of source data (SDV): the inspector shall check the consistency between the eCRF data and the source data, and ask questions if there is any problem.

4. 数据疑问和解答：疑问来源于 EDC 逻辑核查的系统疑问，监查员、数据管理员等人工疑问，研究者需及时解答疑问。数据管理员和监查员进行疑问批复，必要时可再次发出疑问，直至数据"清洁"。

Data questions and answers: The questions come from the system questions of EDC logic check, the manual questions of monitors and data managers, and the researchers need to answer the questions in time. The data administrator and the monitor shall reply to the query, and may issue the query again if necessary, until the data is "clean".

5. 研究者签名：数据录入完成并经 SDV 和数据录入锁定后，研究者进行电子签名审核确认。签名后的如有数据修订，需重新签名。

Signature of the investigator: After the data entry is completed and locked by SDV and data entry, the investigator shall verify and confirm the electronic signature. If there is any data revision after the signature, it shall be re-signed.

6. 数据库锁定：由主要研究者、申办者、统计分析人员和数据管理人员共同签署数据库锁定记录后，数据管理员进行数据库锁定。

Database locking: After the database locking record is signed by the principal investigator, the sponsor, the statistical analyst and the data manager, the data manager locks the database.

7. 数据库提交：数据管理员向统计人员提交数据库。

Database submission: The data manager submits the database to the statistician.

8. eCRF 存档：每个受试者的 eCRF 生成 PDF 电子文档保存。

ECRF archive: eCRF generation PDF electronic document for each subject is saved.

9. 数据管理报告：由数据管理员撰写。

Data Management Report: Written by the data manager.

10. EDC 关闭：统计分析完成后，数据管理员关闭数据库。
EDC shutdown: After the statistical analysis is completed, the data administrator shuts down the database.

（二）记录保存
(II) Record Keeping

伦理委员会、临床试验机构和申办者应该保存临床试验资料的年限如下：
Ethics committees, clinical trial institutions and sponsors should keep clinical trial data for the following years:

1. 伦理委员会应当保留全部有关记录至临床试验完成后至少 10 年；
The ethics committee shall keep all relevant records for at least 10 years after the completion of the clinical trial;

2. 临床试验机构应当保存临床试验资料至临床试验结束后 10 年；
The clinical trial institution shall keep the clinical trial data for 10 years after the completion of the clinical trial;

3. 申办者应当保存临床试验资料至无该医疗器械使用时。
The sponsor shall keep the clinical trial data until the medical device is not used.

九、风险受益分析
IX. Risk and Benefit Analysis

受试者将获得远程手术机器人这一最新技术进行手术，手术机器人手术创伤更小，出血少，操作更加精准，术后康复更快。受试者将获得相应的实验室检查及医疗照顾， 以评价手术的效果。

Subjects will receive the latest technology of remote surgical robots for surgery, which has less trauma, less bleeding, more accurate operation and faster recovery after surgery. Subjects will receive appropriate laboratory tests and medical care to evaluate the results of the procedure.

手术过程中，可能出现试验器械故障，导致受试者受到伤害；手术过程可能出现并发症，如麻醉意外、心脑血管相关意外、大出血、临近器官损伤、血管损伤等；手术后可能出现并发症，如感染、继发出血、肠梗阻等；手术过程中发现受试者不适宜做机器人手术，会为受试者转为传统的腔镜手术或者开放手术。本次临床试验的手术均由临床经验丰富的医生操作，并且在检查和手术过程中遵循指定操作规程。此外，医生将不断接受相应知识、技能的相关培训，在可控范围内，尽可能降低风险的出现，保障受试者的临床安全。

During the operation, the subject may be injured due to the failure of the test instrument; complications may occur during the operation, such as anesthesia accident, cardiovascular and cerebrovascular accidents, massive hemorrhage, adjacent organ injury, vascular injury, etc.; complications may occur after the operation, such as infection,

secondary hemorrhage, intestinal obstruction, etc.; During the operation, it is found that the subject is not suitable for robotic surgery, and the subject will be converted to traditional endoscopic surgery or open surgery. All the operations in this clinical trial were performed by doctors with rich clinical experience, and the specified operating procedures were followed during the examination and operation. In addition, doctors will continue to receive relevant training in knowledge and skills to minimize the occurrence of risks within a controllable range and ensure the clinical safety of subjects.

在正常使用条件下，基于当前技术水平，试验器械带来的受益，所有已知的、可预见的风险以及不良副作用是可接受的。

Under normal conditions of use, based on the current state of the art, the benefits of the investigational device, all known and foreseeable risks, and adverse side effects are acceptable.

十、临床试验的质量控制

X. Quality Control of Clinical Trial

1. 各试验机构应按标准操作规程和质量控制程序进行临床操作；
   All test institutions shall conduct clinical operations in accordance with the standard operating procedures and quality control procedures;

2. 试验开始前，对参与临床试验的研究者进行充分培训，确保研究者充分了解试验流程且熟悉器械使用方法；

Before the start of the trial, the investigators involved in the clinical trial shall be fully trained to ensure that the investigators fully understand the trial process and are familiar with the use of the device;

3. 研究过程中，临床监查员严格按照监查计划进行监查，保证临床试验方案的所有内容都得到严格遵守，研究资料得到及时正确的填写；

During the study, the clinical monitor shall monitor in strict accordance with the monitoring plan to ensure that all the contents of the clinical trial protocol are strictly followed and the study data are timely and correctly filled in;

4. 临床试验数据严格执行数据管理过程。
   Strict data management process for clinical trial data.

十一、临床试验的伦理问题以及知情同意
Ethical Issues in Clinical Trials and Informed Consent

（一）伦理方面的考虑
(I) Ethical Considerations

本次临床试验按照《涉及人的生命科学和医学研究伦理审查办法》、《医疗器械注册与备案管理办法》和《医疗器械临床试验质量管理规范》等法规文件的要求，完全遵循赫尔辛基宣言的伦理学准则，坚持受试者的权益、安全和意志高于研究需要的原则，严格为受试者保密、尊重个人隐私，保证受试者不会因检查结果受到任何歧视或伤害。

In accordance with the requirements of the Regulations on Ethical Review of Life Sciences and Medical Research Involving Human Beings, the Regulations on Registration and Record Management of Medical Devices and the Standards for Quality Management of Clinical Trials of Medical Devices, this clinical trial fully abides by the ethical principles of the Helsinki Declaration and adheres to the rights and interests of subjects. Strictly keep the subject confidential, respect personal privacy, and ensure that the subject will not be discriminated against or harmed by the examination results.

（二）知情同意过程
(II) Informed Consent Process

已根据方案准备好知情同意书文本，具体内容见《知情同意书》样本。

受试者知情同意过程注意事项：

The text of the informed consent form has been prepared in accordance with the protocol, as detailed in the sample Informed Consent Form. Precautions for subject informed consent process:

1. 研究者或委派的负责人执行知情同意过程；
   The investigator or delegated responsible person performs the informed consent process;

2. 知情同意过程应包含与目标受试者做出决定（是否参加临床试验）相关的每个方面；
   The informed consent process should include every aspect related to the target subject's decision (whether to participate in the clinical trial);

3. 应避免对目标受试者强迫、诱导或施加不恰当的影响；
   Avoid forcing, inducing, or exerting inappropriate influence on the target subject;

4. 目标受试者有权保留自己的法律权益；
   Target subjects have the right to retain their legal rights and interests;

5. 使用目标受试者的母语进行易懂的非专业描述，便于目标受试者理解；
   Use the mother tongue of the target subject to make an easy-to-understand non-

professional description, which is easy for the target subject to understand;

6. 提供足够的时间供目标受试者阅读并理解《知情同意书》，并考虑是否参加临床试验；

Provide sufficient time for the target subject to read and understand the Informed Consent Form and consider whether to participate in the clinical trial;

7. 应包含目标受试者及研究者或研究者委派负责人的个人签名；
   It shall include the personal signature of the target subject and the investigator or the person in charge of the investigator;

8. 应给受试者提供一份有签名及日期的《知情同意书》或其它手写资料的复印件；

A signed and dated copy of the Informed Consent Form or other handwritten document should be provided to the subject;

9. 应说明在受试者无法自行处理等特殊情况下如何获得并填写《知情同意书》；
   It shall be explained how to obtain and fill in the Informed Consent Form under special circumstances such as the subject is unable to handle it by himself;

10. 确保整个临床试验过程中将重要的新信息提供给新加入的以及当前的受试者。
   Ensure that important new information is provided to new and current subjects throughout the clinical trial.

十二、对不良事件和器械缺陷报告的规定
XII. Provisions on Adverse Event and Device Defect Reporting

（一）不良事件的定义和报告规定
(I) Definition and Reporting Requirements of Adverse Events

不良事件是指：在医疗器械临床试验过程中出现的不良医学事件，无论是否与试

Adverse events refer to the adverse medical events that occur during the clinical trials of medical devices, regardless of whether they are related to the trial or not.

验医疗器械相关。
Related to medical devices.

医疗器械临床试验中发生不良事件时，研究者应当为受试者提供足够、及时的治疗和处理；当受试者出现并发疾病需要治疗和处理时，研究者应当及时告知受试者。研究者应当记录医疗器械临床试验过程中发生的不良事件。

When an adverse event occurs in the clinical trial of medical devices, the investigator shall provide sufficient and timely treatment and treatment for the subjects; The investigator shall inform the subject in a timely manner when the subject has concurrent diseases requiring treatment and treatment. The investigator shall record the adverse events occurring during the clinical trial of the medical device.

获取不良事件信息：
Obtain adverse event information:

研究者应以简洁的医学术语记录直接观察到的或受试者自发报告的所有不良事件。此外，在每次随访时研究者应使用非诱导的语言向受试者询问不良事件的情况。

The investigator shall record in concise medical terms all adverse events that are directly observed or spontaneously reported by the subject. In addition, the investigator should use non-induced language to ask the subject about the adverse event at each visit.

不良事件记录：
Adverse event record:

记录受试者手术开始后的不良事件。记录的内容应包括：不良事件的发生时间、严重程度、持续时间、采取的措施和转归不良事件严重程度判定标准：

Adverse events after the start of the procedure were recorded. Records shall include: occurrence time, severity, duration, measures taken and criteria for determining the severity of adverse events:

研究者在记录不良事件时，应采用统一的标准对不良事件的严重程度进行描述，不良事件的严重程度分级标准如下：

When recording adverse events, the investigator shall adopt uniform standards to describe the severity of adverse events, and the grading standards for the severity of adverse events are as follows:

轻：患者较容易忍受，或是引起轻微的不适，但不影响日常生活。

中：可引起病人不适，而影响日常生活。

Light: The patient is easy to tolerate, or cause slight discomfort, but does not affect daily life. Middle: May cause the patient to be uncomfortable, but affects the daily life.

重：患者丧失了正常生活的能力，或阻碍了正常的日常生活。
Heavy: The patient loses the ability to live a normal life, or hinders normal daily life.

（二）器械缺陷
(II) Device Defect

器械缺陷，是指临床试验过程中医疗器械在正常使用情况下存在可能危及人体健康和生命安全的不合理风险，如标签错误、质量问题、故障等。

Device defect refers to the unreasonable risks that may endanger human health and life safety under normal use of medical devices in the course of clinical trials, such as label errors, quality problems, failures, etc.

研究者应当记录医疗器械临床试验过程中发现的器械缺陷。

The investigator shall record the device defects found during the clinical trial of the medical device.

（三）严重不良事件的定义
(III) Definition of Serious Adverse Event

严重不良事件，是指医疗器械临床试验过程中发生的导致死亡或者健康状况严重恶化，包括致命的疾病或者伤害、身体结构或者身体功能的永久性缺陷、需要住院治疗或者延长住院时间、需要采取医疗措施以避免对身体结构或者身体功能造成永久性缺陷；导致胎儿窘迫、胎儿死亡或者先天性异常、先天缺损等事件。

Serious adverse event refers to death or serious deterioration of health status occurred during the clinical trial of medical devices, including fatal disease or injury, permanent defect of body structure or body function, need for hospitalization or prolonged hospitalization, and need to take medical measures to avoid permanent defect of body structure or body function; Cause fetal distress, fetal death or congenital abnormalities, congenital defects and other events.

（四）报告程序、联络人信息
(4) Information on Reporting Procedures and Contact Persons

1. 研究者
Researcher

医疗器械临床试验中发生严重不良事件时，研究者应当立即对受试者采取适当的

When serious adverse events occur in the clinical trials of medical devices, the investigator shall immediately take appropriate measures for the subjects.

治疗措施；同时，研究者应当在获知严重不良事件后 24 小时内，向申办者、医疗器械临床试验机构管理部门、伦理委员会报告；并按照临床试验方案的规定随访严重不良事件，提交严重不良事件随访报告；

Treatment measures; at the same time, the investigator shall report to the sponsor, the management department of the medical device clinical trial institution and the ethics committee within 24 hours after being informed of the serious adverse event; Follow up the serious adverse events according to the clinical trial protocol, and submit the follow-up report of serious adverse events;

主要研究者收到申办者提供的试验医疗器械相关严重不良事件和其他安全性信息时，应当及时签收阅读，并考虑受试者的治疗是否进行相应调整，必要时尽早与受试者沟通。

When receiving the serious adverse events and other safety information related to the trial medical device provided by the sponsor, the principal investigator shall sign and read them in time, consider whether the treatment of the subjects should be adjusted accordingly, and communicate with the subjects as soon as possible if necessary.

2. 申办者

Sponsor

申办者应当在获知死亡或者危及生命的临床试验医疗器械相关严重不良事件后 7 日内、获知非死亡或者非危及生命的试验医疗器械相关严重不良事件和其他严重安全性风险信息后 15 日内，向参与临床试验的其他医疗器械临床试验机构、伦理委员会以及主要研究者报告，向申办者所在地省、自治区、直辖市药品监督管理部门报告，向医疗器械临床试验机构所在地省、自治区、直辖市药品监督管理部门和卫生健康管理部门报告，并采取风险控制措施；出现可能影响受试者安全、可能影响医疗器械临床试验实施、可能改变伦理委员会同意意见的信息时，应当及时组织对临床试验方案、知情同意书和其他提供给受试者的信息、以及其他相关文件进行修改，并提交伦理委员会审查；

The sponsor shall, within 7 days after being informed of the death or life-threatening serious adverse events related to the clinical trial medical devices, and within 15 days after being informed of the non-death or non-life-threatening serious adverse events related to the trial medical devices and other serious safety risks, Report to other medical device clinical trial institutions, ethics committees and principal investigators participating in the clinical trial, report to the drug supervision and administration department of the province, autonomous region or municipality directly under the Central Government where the sponsor is located, report to the drug supervision and administration department and the health administration department of the province, autonomous region or municipality directly under the Central Government where the medical device clinical trial institution is located, and take risk control measures; In case of any information that may affect the safety of the subject,

may affect the implementation of the clinical trial of medical devices, or may change the consent of the ethics committee, the clinical trial protocol, the informed consent, other information provided to the subject, and other relevant documents shall be revised in a timely manner and submitted to the ethics committee for review;

出现大范围临床试验医疗器械相关严重不良事件，或者其他重大安全性问题时，申办者应当暂停或者终止医疗器械临床试验，并向所有医疗器械临床试验机构管理部门、伦理委员会以及主要研究者报告，向申办者所在地省、自治区、直辖市药品监督管理部门报告，向所有医疗器械临床试验机构所在地省、自治区、直辖市药品监督管理部门和卫生健康管理部门报告。

In case of serious adverse events or other major safety problems related to medical devices in large-scale clinical trials, the sponsor shall suspend or terminate the clinical trials of medical devices, report to the administrative departments, ethics committees and principal researchers of all medical device clinical trial institutions, and report to the drug supervision and administration departments of the provinces, autonomous regions and municipalities directly under the Central Government where the sponsor is located. Report to the drug supervision and administration departments and health administration departments of the provinces, autonomous regions and municipalities directly under the Central Government where all medical device clinical trial institutions are located.

3. 联络人信息
Contact Information

SAE 报告联系方式
SAE Report Contact

类别 Category	单位 Unit	联系人 Contact person	联系电话 Contact number	邮箱/传真 Mailbox/Fax
申办方 Sponsor	深圳市精锋医疗科 Shenzhen Jingfeng Medical Department 技股份有限公司 Technology Co., Ltd	毛建乐 Mao Jianle	13823125005	mjl@edgemed.cn
药监部门 Drug supervision department 和卫生行 And hygiene 政部门 Administrative Department	参考各省、自治区、直辖市药品监督管理部门和卫生健康主管部门的报告要求。 Refer to the reporting requirements of drug supervision and administration departments and health authorities of provinces, autonomous regions and municipalities directly under the Central Government.			

十三、临床试验方案的偏离与临床试验方案修正的规定
XIII. Provisions on Deviation from and Amendment to the Clinical Trial Protocol

（一）临床试验方案的偏离
(I) Deviation from Clinical Trial Protocol

偏离是指有意或者无意地未遵守医疗器械临床试验方案要求的情形。
Deviation refers to the intentional or unintentional failure to comply with the requirements of the clinical trial protocol of medical devices.

医疗器械临床试验过程中出现临床试验方案的偏离，研究者应记录并分析偏离对受试者权益和安全的可能影响，对医疗器械临床试验的科学性、完整性的可能影响，分为重要方案偏离和一般方案偏离。主要研究者应当按时向伦理委员会报告影响受试者权益和安全的事件或者对临床试验方案的偏离。

If there is any deviation from the clinical trial protocol during the clinical trial of medical devices, the investigator shall record and analyze the possible impact of the deviation on the rights and safety of the subjects, and the possible impact on the scientificity and integrity of the clinical trial of medical devices, which is divided into important protocol deviation and general protocol deviation. The principal investigator shall report the events affecting the rights and safety of the subjects or the deviation from the clinical trial protocol to the ethics committee on time.

重要方案偏离：是指对受试者权益、安全和健康或者临床试验科学性造成显著影响的偏离。

Important protocol deviation: refers to the deviation that has a significant impact on the rights, safety and health of the subjects or the scientificity of the clinical trial.

一般方案偏离：是指对受试者尚未造成实质性影响且不影响试验主次要有效评价的偏离。

General protocol deviation: refers to the deviation that has no substantial impact on the subjects and does not affect the primary and secondary effective evaluation of the trial.

（二）临床试验方案的修正
(II) Amendment of Clinical Trial Protocol

医疗器械临床试验过程中，修订临床试验方案以及知情同意书等文件，应当在获得伦理委员会的书面同意后方可实施。

In the process of clinical trials of medical devices, the revision of clinical trial protocols and informed consent documents shall be carried out only after the

written consent of the ethics committee is obtained.

十四、直接访问源数据、文件
14. Direct Access to Source Data and Files

临床试验的源数据包含纸质和电子两种形式。
The source data of clinical trials include both paper and electronic forms.

纸质源数据主要记录在纸质载体上，分为以下几种：①由研究者或研究者授权人员首次填写产生的数据，例如住院病历、门诊病历等；②受试者亲自填写产生的数据，例如疼痛 VAS 评分表、知情同意书等；③经过验证的受试者电子临床结果评估系统产生的首次以纸质形式呈现的数据，例如：检验科室的检验报告单等；④其他由研究人员或受试者记录的数据，例如临床试验过程中首次产生的原始笔记、备忘录等纸质工作文件；⑤以上源数据的核证副本，例如誊抄在纸质病例报告表中的被主要研究者签字认可的原始数据。

The paper source data is mainly recorded on the paper carrier, which is divided into the following types: ① the data generated by the researcher or the authorized personnel of the researcher for the first time, such as inpatient medical records, outpatient medical records, etc.; ② the data generated by the subjects in person, such as pain VAS score form, informed consent, etc.; ③ The data generated by the electronic clinical outcome evaluation system of the verified subject and presented in paper form for the first time, such as the test report of the laboratory department; ④ Other data recorded by the researcher or the subject, such as the original notes, memos and other paper working documents generated for the first time during the clinical trial; ⑤ Certified copies of the above source data, such as the original data transcribed in the paper case report form and signed by the principal investigator.

电子源数据以电子形式附载于计算机系统中，主要有以下几种：①经过验证的电子数据采集中由研究者现场直接录入产生的数据；②形成检验报告、检查报告等过程

The electronic source data is attached to the computer system in electronic form, mainly including the following types: (1) the data generated by the direct input of the researcher on the spot in the verified electronic data acquisition; ② Process of forming inspection report, inspection report, etc.

中，由经过验证的电子系统直接产生和电子形式存储的，并通过验证的系统传输过程而获得的数据。例如中心实验室的实验室信息管理系统（LIMS）存储了各类实验室指标的检测结果和专业评估报告；医学影像检查科室的医学影像存档和通信系统（PACS）存储了各类医学影像的检查结果和专业评估报告。

Data that is generated directly and stored electronically by a validated electronic system and obtained by transmission through the validated system. For example, the laboratory information management system (LIMS) of the central laboratory stores the test results and professional evaluation reports of various laboratory indicators; the medical image archiving and communication system (PACS) of the medical imaging department stores the test results and professional evaluation reports of various medical images.

所有的源数据/源文件应按照相应的法律法规文件和 SOP 进行合理的保存。例如：纸质源数据文件防水防火；以热敏纸为数据载体的保存应及时存档副本；电子源数据的系统保存年限等问题。

All source data/source documents shall be properly stored in accordance with relevant laws and regulations and SOPs. For example, the paper source data file is waterproof and fireproof; the thermal paper shall be used as the data carrier for storage, and the copy shall be archived in time; The system preservation period of electronic source data and so on.

研究者应具有在其试验机构产生的源数据的控制权。但使用电子病例报告表时，研究者通过授权账户直接控制电子病历报告表/电子数据库中的源数据录入、修改、审阅及签字。在使用纸质病例报告表的情况下，研究者全权监控纸质病历报告表中的源数据，并通过数据澄清表授权数据录入人员对电子数据库中的源数据进行修改。数据文件的控制及访问/核查及审查应设立权限，并且在权限批准情况下进行相应的工作，以保证受试者的权益和避免未经授权的更改。申办者和监管机构应拥有电子源数据的访问和审查权限，访问权限以及访问级别有明确的规定。

The investigator shall have control of the source data generated at his/her testing facility. However, when the electronic case report form is used, the investigator directly controls the entry, modification, review and signature of the source data in the electronic case report form/electronic database through the authorized account. In the case of using the paper case report form, the investigator has full authority to monitor the source data in the paper case report form, and authorize the data entry personnel to modify the source data in the electronic database through the data clarification form. The control and access/verification and review of data files shall be authorized, and the corresponding work shall be carried out under the approval of the authority, so as to ensure the rights and interests of the subjects and avoid unauthorized changes. Sponsors and regulators should have access and review rights to electronic source data, with clearly defined access rights and access levels.

十五、临床试验报告应当涵盖的内容

XV. Contents to Be Covered in the Clinical Trial Report

临床试验报告一般包含医疗器械临床试验基本信息、实施情况、统计分析方法、试验结果、不良事件和器械缺陷报告以及其处理情况、对试验结果的分析讨论、临床试验结论、伦理情况说明、存在问题以及改进建议等内容。

The clinical trial report generally includes the basic information, implementation, statistical analysis methods, test results, adverse events and device defect reports and their handling, analysis and discussion of the test results, clinical trial conclusions, ethical description, existing problems and suggestions for improvement of medical devices.

十六、保密原则
XVI. Confidentiality

所有在试验中收集到的受试者的信息都将根据法律规定的程度进行保密。临床试验相关资料中以受试者编号和受试者姓名拼音缩写作为受试者识别的主要依据；受试者个人资料记录在受试者鉴认编码表中，受试者鉴认编码表由主要研究者作为保密资料保存。受试者的个人信息在没有受试者的书面许可的情况下不会进行公布。伦理委员会、国家药品监督管理局或卫生健康委员会主管部门或者申办者在工作需要时按照规定程序可以查询受试者参加试验的个人资料。此项试验的内容有可能发表，所发表的内容不会包含任何受试者的个人信息。

All subject information collected during the trial will be kept confidential to the extent required by law. In the relevant data of the clinical trial, the subject number and the phonetic abbreviation of the subject's name are used as the main basis for subject identification; The personal data of the subjects were recorded in the subject identification coding table, which was kept as confidential data by the principal investigator. The subject's personal information will not be released without the subject's written permission. The ethics committee, the National Medical Products Administration or the competent department of the health committee or the sponsor may inquire about the personal data of the subjects participating in the trial in accordance with the prescribed procedures when necessary. The content of this trial is likely to be published and will not contain any personal information about the subject.

十七、各方承担的职责
XVII. Responsibilities of the Parties

（一）申办者职责
(I) Responsibilities of the Applicant

1. 申办者应当对医疗器械临床试验的真实性、合规性负责。
The sponsor shall be responsible for the authenticity and compliance of the clinical trial of medical devices.

2. 申办者的质量管理体系应当覆盖医疗器械临床试验的全过程，包括医疗器械临床试验机构和主要研究者的选择、临床试验方案的设计、医疗器械临床试验的实施、记录、结果报告和文件归档等。申办者的质量管理措施应当与临床试验的风险相适应。

The quality management system of the sponsor shall cover the whole process of medical device clinical trials, including the selection of medical device clinical trial institutions and principal investigators, the design of clinical trial schemes, the implementation, record, result report and document filing of medical device clinical trials. The sponsor's quality management measures should be appropriate to the risks of the clinical trial.

3. 申办者发起医疗器械临床试验前应当：
Before initiating a clinical trial of a medical device, the sponsor shall:

(1) 确保产品设计已定型，完成试验医疗器械的临床前研究，包括性能验证以及确认、基于产品技术要求的产品检验报告、风险受益分析等，且结果应当能够支持该项医疗器械临床试验；

Ensure that the product design has been finalized, complete the pre-clinical study of the trial medical device, including performance verification and validation, product inspection report based on product technical requirements, risk and benefit analysis, etc., and the results should be able to support the clinical trial of the medical device;

(2) 根据试验医疗器械的特性，选择已备案的医疗器械临床试验机构、专业和主要研究者；

Select the registered medical device clinical trial institution, specialty and principal investigator according to the characteristics of the trial medical device;

(3) 负责组织制定研究者手册、临床试验方案、知情同意书、病例报告表、标准操作规程以及其他相关文件，并向医疗器械临床试验机构和主要研究者提供。

Be responsible for organizing the preparation of the investigator's manual, clinical trial protocol, informed consent, case report form, standard operating procedures and other relevant documents, and providing them to the medical device clinical trial institution and the principal investigator.

4. 申办者应当与医疗器械临床试验机构和主要研究者签订合同，明确各方在医疗器械临床试验中的权利和义务。

The sponsor shall sign a contract with the medical device clinical trial institution and the principal investigator to clarify the rights and obligations of each party in the medical device clinical trial.

5. 申办者应当在医疗器械临床试验经伦理审查通过并且与医疗器械临床试验机构签订合同后，向申办者所在地省、自治区、直辖市药品监督管理部门进行临床试验项目备案。

The sponsor shall file the clinical trial project with the drug supervision and administration department of the province, autonomous region or municipality directly under the Central Government where the sponsor is located after the clinical trial of medical devices has passed the ethical review and signed a contract with the clinical trial institution of medical devices.

6. 医疗器械临床试验备案完成后，该医疗器械临床试验机构方可开始第一例受试者知情同意以及筛选。

After the medical device clinical trial filing is completed, the medical device clinical trial institution can start the informed consent and screening of the first subject.

7. 医疗器械临床试验开始前，申办者应当负责组织与该医疗器械临床试验相关的培训，如试验医疗器械的原理、适用范围、产品性能、操作方法、安装要求、技术指标以及临床试验方案、标准操作规程以及其他相关文件等。

Before the start of the clinical trial of medical devices, the sponsor shall be responsible for organizing the training related to the clinical trial of medical devices, such as the principle, scope of application, product performance, operation methods, installation requirements, technical indicators, clinical trial scheme, standard operating procedures and other relevant documents of the trial medical devices.

8. 申办者应当免费提供试验医疗器械，并符合以下要求：

The sponsor shall provide the trial medical device free of charge and meet the following requirements:

(1) 试验医疗器械应当按照医疗器械生产质量管理规范的相关要求生产且质量合格；

The trial medical devices shall be manufactured in accordance with the relevant requirements of the Good Manufacturing Practice for Medical Devices and shall be qualified;

(2) 确定试验医疗器械的运输条件、储存条件、有效期等；

Determine the transportation conditions, storage conditions, validity period, etc. of the test medical device;

(3) 试验医疗器械应当按照临床试验方案要求进行适当包装和保存；包装标签上应当标明产品信息，具有易于识别、正确编码的标识，标明仅用于医疗器械临床试验；

The trial medical devices shall be properly packaged and preserved in accordance with the requirements of the clinical trial scheme; The product information shall be marked on the package label, which shall be easily identified and correctly coded, and shall be used only for clinical trials of medical devices;

(4) 医疗器械临床试验获得伦理委员会同意后，申办者负责在规定的条件下将试验医疗器械运输至医疗器械临床试验机构；

After the medical device clinical trial is approved by the ethics committee, the sponsor is responsible for transporting the trial medical device to the medical device clinical trial institution under the specified conditions;

(5) 对从医疗器械临床试验机构回收的试验医疗器械，申办者负责保存回收处置等记录。

For the trial medical devices recovered from the medical device clinical trial institution, the sponsor shall be responsible for keeping the records of recovery and disposal.

9. 受试者发生与医疗器械临床试验相关的损害或者死亡时，申办者应当承担相应的治疗费用、补偿或者赔偿，但不包括研究者和医疗器械临床试验机构自身过失以及受试者自身疾病进展所致的损害。

In case of injury or death related to the clinical trial of medical devices, the sponsor shall bear the corresponding treatment expenses, compensation or compensation, but excluding the injury caused by the negligence of the investigator and the clinical trial institution of medical devices and the disease progression of the subject.

10. 申办者应当负责医疗器械试验期间安全性信息的评估和报告：

The sponsor shall be responsible for the evaluation and reporting of safety information during the trial of the medical device:

(1) 申办者应当在获知死亡或者危及生命的临床试验医疗器械相关严重不良事件后 7 日内、获知非死亡或者非危及生命的试验医疗器械相关严重不良事件和其他严重安全性风险信息后 15 日内，向参与临床试验的其他医疗器械临床试验机构、伦理委员会以及主要研究者报告，向申办者所在地省、自治区、直辖市药品监督管理部门报告，向医疗器械临床试验机构所在地省、自治区、直辖市药品监督管理部门和卫生健康管理部门报告，并采取风险控制措施；出现可能影响受试者安全、可能影响医疗器械临床试验实施、可能改变伦理委员会同意意见的信息时，应当及时组织对临床试验方案、知情同意书和其他提供给受试者的信息、以及其他相关文件进行修改，并提交伦理委员会审查；

The sponsor shall, within 7 days after being informed of the death or life-threatening

serious adverse events related to the clinical trial medical devices, and within 15 days after being informed of the non-death or non-life-threatening serious adverse events related to the trial medical devices and other serious safety risks, Report to other medical device clinical trial institutions, ethics committees and principal investigators participating in the clinical trial, report to the drug supervision and administration department of the province, autonomous region or municipality directly under the Central Government where the sponsor is located, report to the drug supervision and administration department and the health administration department of the province, autonomous region or municipality directly under the Central Government where the medical device clinical trial institution is located, and take risk control measures; In case of any information that may affect the safety of the subject, may affect the implementation of the clinical trial of medical devices, or may change the consent of the ethics committee, the clinical trial protocol, the informed consent, other information provided to the subject, and other relevant documents shall be revised in a timely manner and submitted to the ethics committee for review;

(2) 出现大范围临床试验医疗器械相关严重不良事件，或者其他重大安全性问题时，申办者应当暂停或者终止医疗器械临床试验，并向所有医疗器械临床试验机构管理部门、伦理委员会以及主要研究者报告，向申办者所在地省、自治区、直辖市药品监督管理部门报告，向所有医疗器械临床试验机构所在地省、自治区、直辖市药品监督管理部门和卫生健康管理部门报告。

In case of serious adverse events or other major safety problems related to medical devices in large-scale clinical trials, the sponsor shall suspend or terminate the clinical trials of medical devices, report to the administrative departments, ethics committees and principal researchers of all medical device clinical trial institutions, and report to the drug supervision and administration departments of the provinces, autonomous regions and municipalities directly under the Central Government where the sponsor is located. Report to the drug supervision and administration departments and health administration departments of the provinces, autonomous regions and municipalities directly under the Central Government where all medical device clinical trial institutions are located.

11. 申办者应当承担医疗器械临床试验监查责任，制定监查标准操作规程，并选择符合要求的监查员履行监查职责：

The sponsor shall be responsible for the supervision of clinical trials of medical devices, formulate the standard operating procedures for supervision, and select qualified supervisors to perform the supervision duties:

(1) 监查员人数以及监查次数应当与医疗器械临床试验的复杂程度和参与临床试验的医疗器械临床试验机构数量相匹配；

The number of inspectors and the number of inspections shall match the complexity of the clinical trial of medical devices and the number of medical device clinical trial institutions participating in the clinical trial;

(2) 监查员应当受过相应的培训，熟悉本规范和相关法律法规，具备相关专业背景知识，熟悉试验医疗器械的相关研究资料和同类产品临床方面的信息、临床试验方案以及其相关的文件，能够有效履行监查职责；

The supervisor shall have received corresponding training, be familiar with this specification and relevant laws and regulations, have relevant professional background knowledge, be familiar with relevant research data of the trial medical device and clinical information of similar products, clinical trial scheme and relevant documents, and be able to effectively perform supervision duties;

(3) 监查员应当遵守由申办者制定的监查标准操作规程，督促医疗器械临床试验按照临床试验方案实施。监查的内容包括医疗器械临床试验机构和研究者在临床试验实施过程中对临床试验方案、本规范和相关法律法规的依从性；受试者知情同意书签署、筛选、随访、权益和安全保障；试验医疗器械和对照医疗器械（如适用）的管理和使用；生物样本的管理和使用（如适用）；不良事件和器械缺陷的处理；安全性信息的报告；临床试验数据记录以及病例报告表填写等。

The monitor shall abide by the standard operating procedures for monitoring formulated by the sponsor, and supervise the implementation of the clinical trial of medical devices in accordance with the clinical trial scheme. The contents of the audit include the compliance of the medical device clinical trial institution and the investigator with the clinical trial protocol, this specification and relevant laws and regulations during the implementation of the clinical trial; the signing, screening, follow-up, rights and interests, and safety guarantee of the subject's informed consent; Management and use of trial medical devices and control medical devices (if applicable); management and use of biological samples (if applicable); handling of adverse events and device defects; reporting of safety information; recording of clinical trial data and filling of case report forms, etc.

12. 为保证临床试验的质量，申办者可以组织独立于医疗器械临床试验、有相应培训和经验的稽查员对临床试验实施情况进行稽查，评估临床试验是否符合临床试验方案、本规范和相关法律法规的规定。

To ensure the quality of the clinical trial, the sponsor may organize inspectors who are independent of the clinical trial of medical devices and have corresponding training and experience to inspect the implementation of the clinical trial and evaluate whether the clinical trial conforms to the clinical trial scheme, this specification and relevant laws and regulations.

13. 申办者应当确保医疗器械临床试验的实施遵守临床试验方案，发现医疗器械临床试验机构和研究者不遵守临床试验方案、本规范和相关法律法规的，应当及时指出并予以纠正；如情况严重或者持续不改，应当终止该临床试验机构和研究者继续参加该临床试验，并书面向临床试验机构所在地省、自治区、直辖市药品监督管理部门报告。

The sponsor shall ensure that the implementation of the clinical trial of medical devices complies with the clinical trial scheme, and shall promptly point out and correct the non-compliance of the clinical trial scheme, this specification and relevant laws and regulations by the clinical trial institutions and researchers of medical devices; If the circumstances are serious or persist, the clinical trial institution and the investigator shall be terminated to continue to participate in the clinical trial, and a written report shall be made to the drug regulatory department of the province, autonomous region or municipality directly under the Central Government where the clinical trial institution is located.

14. 申办者应当在医疗器械临床试验暂停、终止或者完成后 10个工作日内，书面报告所有的主要研究者、医疗器械临床试验机构管理部门、伦理委员会。

The sponsor shall, within 10 working days after the suspension, termination or completion of the clinical trial of the medical device, report in writing to all the principal investigators, the administrative department of the clinical trial institution of the medical device and the ethics committee.

15. 申办者应当在医疗器械临床试验终止或者完成后 10个工作日内，向申办者所在地省、自治区、直辖市药品监督管理部门报告。

The sponsor shall report to the drug regulatory department of the province, autonomous region or municipality directly under the Central Government where the sponsor is located within 10 working days after the termination or completion of the clinical trial of medical devices.

（二）临床试验机构和研究者职责

(II) Responsibilities of Clinical Trial Institutions and Researchers

1. 医疗器械临床试验机构应当符合备案条件，建立临床试验管理组织架构和管理制度。医疗器械临床试验机构应当具有相应的临床试验管理部门，承担医疗器械临床试验的管理工作。

Medical device clinical trial institutions shall meet the requirements for filing, and establish the organizational structure and management system for clinical trial management. A medical device clinical trial institution shall have a corresponding clinical trial management department to undertake the management of medical device clinical trials.

2. 医疗器械临床试验机构管理部门应当负责在医疗器械临床试验机构备案管理信息系统中填报、管理和变更医疗器械临床试验机构备案信息，包括临床试验专业、

The administrative department of the medical device clinical trial institution shall be responsible for filling in, managing and changing the filing information of the medical device clinical trial institution in the filing management information system of the medical device clinical trial institution, including the clinical trial specialty,

主要研究者等信息；负责在备案系统中在线提交上一年度实施医疗器械临床试验工作总结报告；负责在伦理委员会对医疗器械临床试验审查前，组织评估该临床试验主要研究者的资质并完成其备案。

Principal investigator and other information; be responsible for submitting the summary report on the implementation of clinical trials of medical devices in the previous year online in the filing system; Be responsible for organizing the evaluation of the qualification of the principal investigator of the clinical trial and completing its filing before the review of the clinical trial of medical devices by the ethics committee.

3. 医疗器械临床试验机构应当建立质量管理制度，涵盖医疗器械临床试验实施的全过程，包括培训和考核、临床试验的实施、医疗器械的管理、生物样本的管理、不良事件和器械缺陷的处理以及安全性信息的报告、记录、质量控制等制度，确保主要研究者履行其临床试验相关职责，保证受试者得到妥善的医疗处理，确保试验产生数据的真实性。

Medical device clinical trial institutions shall establish a quality management system covering the whole process of the implementation of medical device clinical trials, including training and assessment, implementation of clinical trials, management of medical devices, management of biological samples, handling of adverse events and device defects, and reporting, recording and quality control of safety information. To ensure that the principal investigator performs his or her duties related to the clinical trial, to ensure that the subjects receive proper medical treatment, and to ensure the authenticity of the data generated by the trial.

4. 医疗器械临床试验机构在接受医疗器械临床试验前，应当根据试验医疗器械的特性评估相关资源，确保具备相匹配的资质、人员、设施、条件等。

Before accepting the clinical trial of medical devices, the medical device clinical trial institution shall evaluate the relevant resources according to the characteristics of the trial medical devices to ensure that it has the matching qualifications, personnel, facilities and conditions.

5. 医疗器械临床试验机构和研究者应当配合申办者组织的监查和稽查，以及药品监督管理部门、卫生健康管理部门开展的检查。

Medical device clinical trial institutions and researchers shall cooperate with the supervision and inspection organized by the sponsor, as well as the inspection carried out by the drug supervision and administration department and the health administration department.

6. 医疗器械临床试验机构应当按照相关法律法规和与申办者的合同，妥善保存临床试验记录和基本文件。

The medical device clinical trial institution shall properly keep the clinical trial records and basic documents in accordance with relevant laws and regulations and the contract with the sponsor.

7. 负责医疗器械临床试验的主要研究者应当具备下列条件：
The principal investigator in charge of the clinical trial of medical devices shall meet the following requirements:

(1) 已完成医疗器械临床试验主要研究者备案；
The principal investigator of the clinical trial of medical devices has been put on record;

(2) 熟悉本规范和相关法律法规；
Be familiar with this specification and relevant laws and regulations;

(3) 具有试验医疗器械使用所要求的专业知识和经验，经过临床试验相关培训，有临床试验的经验，熟悉申办者所提供的医疗器械临床试验方案、研究者手册等资料；

Have the professional knowledge and experience required for the use of trial medical devices, have received relevant training in clinical trials, have experience in clinical trials, and be familiar with the clinical trial scheme of medical devices, the investigator's manual and other materials provided by the sponsor;

(4) 有能力协调、支配和使用进行该项医疗器械临床试验的人员和设备，且有能力处理医疗器械临床试验中发生的不良事件和其他关联事件。

Have the ability to coordinate, control and use the personnel and equipment for the clinical trial of the medical device, and have the ability to deal with the adverse events and other related events occurring in the clinical trial of the medical device.

8. 主要研究者应当确保医疗器械临床试验遵守伦理委员会同意的最新版本临床试验方案；在约定的时限内，按照本规范和相关法律法规的规定实施医疗器械临床试验。

The principal investigator shall ensure that the clinical trial of the medical device complies with the latest version of the clinical trial protocol approved by the ethics committee; The clinical trial of medical devices shall be carried out within the agreed time limit in accordance with the provisions of this specification and relevant laws and regulations.

9. 主要研究者可以根据医疗器械临床试验的需要，授权经过临床试验相关培训的研究者，组织进行受试者招募和知情同意、筛选和随访；试验医疗器械和对照医疗器械（如适用）的管理和使用；生物样本的管理和使用（如适用）；不良事件和器械缺陷的处理；临床试验数据记录以及病例报告表填写等。

The principal investigator may authorize the investigators who have received relevant training in clinical trials to organize subject recruitment, informed consent, screening and follow-up according to the needs of clinical trials of medical devices; Management and use of trial medical devices and control medical devices (if applicable); management and use of biological samples (if applicable); handling of adverse events and device defects; clinical trial data recording and case report form filling, etc.

10. 参与医疗器械临床试验的研究者应当：
An investigator participating in a clinical trial of a medical device shall:

(1) 具有承担医疗器械临床试验相应的专业技术资格、培训经历和相关经验；
Have the corresponding professional and technical qualifications, training experience and relevant experience in undertaking clinical trials of medical devices;

(2) 参加申办者组织的与该医疗器械临床试验相关的培训，并在主要研究者授权的范围内参与医疗器械临床试验；

Participate in the training related to the clinical trial of the medical device organized by the sponsor, and participate in the clinical trial of the medical device within the scope authorized by the principal investigator;

(3) 熟悉试验医疗器械的原理、适用范围或者预期用途、产品性能、操作方法、安装要求以及技术指标等，了解该试验医疗器械临床前研究相关资料；

Be familiar with the principle, scope of application or intended use, product performance, operation method, installation requirements and technical indicators of the test medical device, and understand the relevant data of the preclinical study of the test medical device;

(4) 充分了解并且遵守临床试验方案、本规范和相关法律法规规定以及与医疗器械临床试验相关的职责；

Fully understand and comply with the clinical trial protocol, this specification and relevant laws and regulations, as well as the responsibilities related to the clinical trial of medical devices;

(5) 掌握临床试验可能产生风险的防范以及紧急处理方法。
Grasp the prevention and emergency treatment methods of possible risks in clinical trials.

11. 研究者应当遵守《世界医学大会赫尔辛基宣言》的伦理准则及相关伦理要求，并符合以下要求：

The investigator shall comply with the ethical guidelines and related ethical requirements of the World Medical Congress Declaration of Helsinki and meet the following requirements:

(1) 应当使用经伦理委员会同意的最新版本知情同意书和其他提供给受试者的信息；

The latest version of the informed consent form approved by the ethics committee and other information provided to the subject should be used;

(2) 在受试者参与临床试验前，应当向受试者说明试验医疗器械以及临床试验有关的详细情况，告知受试者可能的受益和已知的、可以预见的风险，经充分和详细解释后由受试者在知情同意书上签署姓名和日期，研究者在知情同意书上应当签署姓名和日期；

Before the subject participates in the clinical trial, the subject shall be informed of the details of the trial medical device and the clinical trial, the possible benefits and the known and foreseeable risks. After full and detailed explanation, the subject shall sign the name and date on the informed consent, and the researcher shall sign the name and date on the informed consent.

(3) 受试者为无民事行为能力人或者限制民事行为能力人的，应当依法获得其监护人的书面知情同意；受试者缺乏阅读能力的，应当有一位公正见证人见证整个知情同意过程并在知情同意书上签字并注明日期；

If the subject is a person without or with limited capacity for civil conduct, he shall obtain the written informed consent of his guardian according to law. If the subject lacks the ability to read, an impartial witness should witness the whole process of informed consent and sign and date the informed consent form.

(4) 不应当强迫或者以其他不正当方式诱使受试者参加临床试验；

The subject shall not be forced or induced to participate in the clinical trial by other improper means;

(5) 确保知情同意书更新并获得伦理委员会审查同意后，所有受影响的未结束试验流程的受试者，都签署新修订的知情同意书。

Ensure that all affected subjects who have not completed the trial process sign the newly revised informed consent form after the updated informed consent form has been reviewed and approved by the ethics committee.

12. 研究者对申办者提供的试验医疗器械和对照医疗器械（如适用）有管理责任，应当确保其仅用于参加该医疗器械临床试验的受试者，在临床试验期间按照要求储存和保管，在临床试验完成或者终止后按照相关法律法规和与申办者的合同进行处理。

The investigator shall be responsible for the management of the trial medical device and the control medical device (if applicable) provided by the sponsor, and shall ensure that they are only used for the subjects participating in the clinical trial of the medical device, stored and kept as required during the clinical trial, and handled in accordance with relevant laws and regulations and the contract with the sponsor after the completion or termination of the clinical trial.

13. 研究者应当确保医疗器械临床试验中生物样本的采集、处理、保存、运输、销毁等符合临床试验方案和相关法律法规。

The investigator shall ensure that the collection, processing, storage, transportation and destruction of biological samples in the clinical trial of medical devices comply with the clinical trial protocol and relevant laws and regulations.

14. 医疗器械临床试验中发生不良事件时，研究者应当为受试者提供足够、及时的治疗和处理；当受试者出现并发疾病需要治疗和处理时，研究者应当及时告知受试者。研究者应当记录医疗器械临床试验过程中发生的不良事件和发现的器械缺陷。

When an adverse event occurs in the clinical trial of medical devices, the investigator shall provide sufficient and timely treatment and treatment for the subjects; The investigator shall inform the subject in a timely manner when the subject has concurrent diseases requiring treatment and treatment. The investigator shall record the adverse events and device defects found during the clinical trial of the medical device.

15. 研究者应当及时报告医疗器械临床试验中的安全性信息：

The investigator shall report the safety information in the clinical trial of medical devices in a timely manner:

(1) 医疗器械临床试验中发生严重不良事件时，研究者应当立即对受试者采取适当的治疗措施；同时，研究者应当在获知严重不良事件后 24 小时内，向申办者、医疗器械临床试验机构管理部门、伦理委员会报告；并按照临床试验方案的规定随访严重不良事件，提交严重不良事件随访报告；

When a serious adverse event occurs in the clinical trial of medical devices, the investigator shall immediately take appropriate treatment measures for the subjects; meanwhile, the investigator shall report to the sponsor, the administrative department of the clinical trial institution of medical devices and the ethics committee within 24 hours after being informed of the serious adverse event; Follow up the serious adverse events according to the clinical trial protocol, and submit the follow-up report of serious adverse events;

(2) 发现医疗器械临床试验的风险超过可能的受益,需要暂停或者终止临床试验时，主要研究者应当向申办者、医疗器械临床试验机构管理部门、伦理委员会报告,及时通知受试者，并保证受试者得到适当治疗和随访。

If it is found that the risk of the clinical trial of medical devices exceeds the possible benefit and the clinical trial needs to be suspended or terminated, the principal investigator shall report to the sponsor, the administrative department of the clinical trial institution of medical devices and the ethics committee, notify the subjects in time, and ensure that the subjects receive appropriate treatment and follow-up.

16. 主要研究者应当对收到的安全性信息及时处理：

The Principal Investigator shall promptly process the safety information received:

(1) 收到申办者提供的试验医疗器械相关严重不良事件和其他安全性信息时，应当及时签收阅读，并考虑受试者的治疗是否进行相应调整，必要时尽早与受试者沟通；

When receiving the serious adverse events and other safety information related to the trial medical device provided by the sponsor, the applicant shall sign and read them in time, consider whether the treatment of the subject is adjusted accordingly, and communicate with the subject as soon as possible if necessary;

(2) 收到申办者或者伦理委员会需要暂停或者终止医疗器械临床试验的通知时，

应当及时通知受试者，并保证受试者得到适当治疗和随访。

When receiving the notice of suspending or terminating the clinical trial of medical devices from the sponsor or the ethics committee, the subject shall be notified in a timely manner, and the subject shall be guaranteed to receive appropriate treatment and follow-up.

17. 主要研究者应当按时向伦理委员会报告医疗器械临床试验的进展，及时报告影响受试者权益和安全的事件或者对临床试验方案的偏离。

The principal investigator shall report the progress of the clinical trial of medical devices to the ethics committee on time, and report the events affecting the rights and safety of the subjects or the deviation from the clinical trial protocol in a timely manner.

18. 医疗器械临床试验机构和研究者对申办者严重或者持续违反本规范和相关法律法规，或者要求改变试验数据、结论的行为，应当书面向申办者所在地省、自治区、直辖市药品监督管理部门报告。

Medical device clinical trial institutions and researchers shall report in writing to the drug supervision and administration department of the province, autonomous region or municipality directly under the Central Government where the sponsor is located if the sponsor seriously or continuously violates the Code and relevant laws and regulations, or requests to change the test data and conclusions.

附录 1 术后 Clavien-Dindo 系统分级
Appendix 1 Grading of Clavien-Dindo System After Operation

分级<br>Grading	定义<br>Definition
1 级<br>Level 1	术后常见原因导致的不需要药物、手术、内镜及放射干预的临床症状。允许适当的治疗包括：止吐药、解热药、镇痛药、利尿药、电解质类及物理治疗。也包括床边打开感染的切口。<br>Clinical signs and symptoms due to common postoperative causes that do not require medical, surgical, endoscopic, or radiological intervention. Allow appropriate treatment including: antiemetics, antipyretics, analgesics, diuretics, electrolytes, and physical therapy. It also involves opening infected incisions at the bedside.
2 级<br>Level 2	需要除 I 级干预药物以外的药物治疗。包括输血和全肠外营养。<br>Medications other than class I intervention drugs are required. Including blood transfusion and total parenteral nutrition.
3 级<br>Level 3	需要手术、内镜及放射干预<br>Requires surgical, endoscopic, and radiological intervention
3a 级<br>Grade 3A	不需要在基础麻醉下进行的干预措施<br>Interventions that do not require basal anesthesia
3b 级<br>Level 3B	需要在基础麻醉下进行的干预措施<br>Interventions Needed Under Basal Anesthesia
4 级<br>Level 4	威胁生命的并发症，包括中枢神经系统并发症*，需要进入 ICU 治疗。<br>Life-threatening complications, including central nervous system complications *, require admission to the ICU.
4a 级<br>Grade 4A	单器官功能障碍（包括透析）<br>Single organ dysfunction (including dialysis)
4b 级<br>Class 4B	多器官功能障碍<br>Multiple Organ Dysfunction
5 级<br>Level 5	患者死亡<br>The patient died
后缀“d”<br>Suffix "d"	如果患者出院时仍然有并发症的症状，需要在各等级的并发症后添加后缀“d”以示“未治愈”。这些添加后缀的患者需要进一步随访，以全面评估并发症。<br>If the patient is discharged with symptoms of a complication, the suffix "d" is added to each grade of complication to indicate "not cured.". These suffixed patients require further follow-up to fully assess for complications.

*中枢神经系统并发症包括：脑出血、缺血性中风、蛛网膜下腔出血，但不包括短暂性缺血发作（TIA）。

* CNS complications include: cerebral hemorrhage, ischemic stroke, subarachnoid hemorrhage, but not transient ischemic attack (TIA).

附录 2 **视觉模拟评分表（Visual Analogue Scale/Score VAS）**
Appendix 2 Visual Analogue Scale (Visual Analogue Scale/Score VAS)

受试者筛选号：
Subject screening number:

受试者签名：
Subject's signature:

日期:　　年　　月　　日
Dat:　　Year　Month　Day

以下评分表是为了帮助医护人员了解您手术后的疼痛情况，请您对此时手术部位的疼痛情况做出真实的回答。以下是疼痛评分说明，请仔细阅读，然后做出选择。

The following score sheet is designed to help healthcare workers understand your pain after surgery. Please give a true answer to your pain at the surgical site at this time. Here are the pain score instructions, read them carefully and make your choice.

在纸上面划一条 10cm 的横线，横线的一端为 0，表示无痛；另一端为 10，表示剧痛；中间部分表示不同程度的疼痛。请根据自我感觉在对应的数字刻度上标记"X"，表示疼痛的程度。

Draw a 10 cm horizontal line on the paper. One end of the horizontal line is 0, indicating no pain; the other end is 10, indicating severe pain; The middle section indicates varying degrees of pain. Please mark "X" on the corresponding digital scale according to your own feeling, indicating the degree of pain.

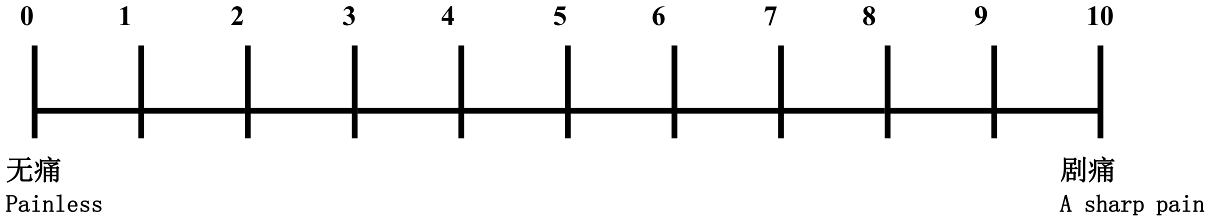

0：无痛，无任何疼痛感觉；
0: No pain, no pain feeling;

1-3：轻度疼痛，不影响工作，生活；
1-3: Mild pain, not affecting work and life;

4-6：中度疼痛，影响工作，不影响生活；
4-6: Moderate pain, affecting work, not affecting life;

7-10：重度疼痛，疼痛剧烈，影响工作及生活。
7-10: Severe pain, severe pain, affecting work and life.

附录 3 NASA-TLX 量化表
Appendix 3 NASA-TLX Quantification Table

受试者筛选号：
Subject screening number:

医生姓名：
Doctor's name:

本研究采用NASA-TLX（NASA_Task Load Index）量表对主刀医生操作胸腹腔内窥镜手术系统后的主观疲劳感和手术完成满意度进行评估，每一项评估指标划分为 21 个不同等级。

In this study, NASA-TLX (NASA _ Task Load Index) scale was used to evaluate the subjective fatigue and satisfaction of surgeons after operating the thoracoabdominal endoscopic surgery system, and each evaluation index was divided into 21 different levels.

1. 脑力需求（完成任务过程中付出多少脑力活动？如思考、决定、计算、记忆、观察、搜查等。该工作从脑力方面对你而言是容易还是困难？简单还是复杂？要求严格还是不严格？）
   Mental demand (how much mental activity is involved in completing a task, such as thinking, deciding, calculating, remembering, observing, searching, etc. Is the job mentally easy or difficult for you? Simple or complex? Strict or not?

非常低（1） 非常高（21）
Very Low (1) Very high (21)

2. 体力需求（完成任务过程中需付出多少体力？如：拖拽、旋转、控制、进行活动的程度等。该任务从体力方面对你而言是容易还是困难？是缓慢还是快速？肌肉感到松弛还是紧张？动作轻松还是费力？）
   Physical demands (how much physical effort is required to complete the task? E.g., dragging, rotation, control, degree of activity performed, etc. Is the task physically easy or difficult for you? Is it slow or fast? Does the muscle feel relaxed or tense? Is the movement easy or strenuous?

非常低（1） 非常高（21）
Very Low (1) Very high (21)

3. 时间需求（工作速率或节奏是缓慢并使人感到从容不迫，还是快速令人感到慌乱？）

   Time requirements (is the rate or pace of work slow and leisurely, or is it fast and disconcerting?)

非常低（1） 非常高（21）
Very Low (1) Very high (21)

4. 业绩水平（完成目标取得的成绩怎么样？对取得的成绩，您的满意程度有多大？）

   Performance level (what is the achievement of the goal? How satisfied are you with the achievement?)

非常低（1） 非常高（21）
Very Low (1) Very high (21)

5. 努力程度（你付出了多少努力来完成任务？）

Effort (how much effort did you put into the task?)

非常低（1） 非常高（21）
Very Low (1) Very high (21)

6. 受挫程度（在执行任务时，你感到不安，沮丧、急躁、烦恼的程度有多大？）
How frustrated are you (how upset, frustrated, irritated, or annoyed are you with the task?)

非常低（1） 非常高（21）
Very Low (1) Very high (21)

附录 4 **远程手术操作评分表**

Appendix 4 Score Sheet of Remote Surgical Operation

受试者筛选号：____________主刀医生：________________ 日期：________________

评分规则：5 分：非常满意，能够满足临床所有的需求。4 分：比较满意，能够满足临床基本的需求。3 分：基本满意，能够满足临床最低的需求。2 分：不太满意，不能满足临床最低的需求。1 分：很不满意，完全不能进行临床操作。

Subject screening number: Chief surgeon: Dat:

Scoring rule: 5 points: Very satisfied, able to meet all clinical needs. 4 points: relatively satisfactory, able to meet the basic clinical needs. 3 points: basically satisfied, able to meet the minimum clinical needs. 2 points: Not satisfied, unable to meet the minimum clinical needs. 1 point: Very dissatisfied, completely unable to perform clinical procedures.

序号<br>Serial number	一级指标<br>Level 1 indicator	二级指标<br>Secondary indicators	评分<br>Rating
1	延迟稳定指标<br>Delayed stability indicator	内窥镜图像延迟<br>Endoscopic image delay	
2		主从操作延迟<br>Master-slave operation delay	
3		成像稳定性，无卡顿<br>Imaging stability, no stutter	
4		主从操作稳定，无卡断<br>The master-slave operation is stable without jamming.	
5		总体评价<br>Overall evaluation	
6	声音传输质量<br>Sound transmission quality	声音传输即时性<br>The immediacy of sound transmission	
7		声音传输稳定性<br>Sound transmission stability	
8		总体评价<br>Overall evaluation	
9	内窥镜图像质量<br>Endoscopic image quality	视野大小<br>Field of view size	
10		清晰度<br>Clarity	
11		景深<br>Depth of field	
12		分辨能力<br>Resolving power	
13		立体感<br>Three-dimensional sense	
14		镜头抗模糊的能力<br>The ability of the lens to resist blurring	
15		抗反光的能力<br>Anti-reflection ability	
16		图像/色彩保真性	

		Image/color fidelity	
17		总体评价<br>Overall evaluation	
18	手术操作<br>Operative procedure	器械操作范围<br>Scope of device operation	
19		器械灵活性<br>Device flexibility	
20		器械夹持<br>Instrument clamping	
21		器械剪切力<br>Device shear force	
22		器械电切<br>Instrumental electrotomy	
23		器械电凝<br>Instrument electrocoagulation	
24		器械运动延迟性<br>Device motion retardation	
25		器械精准度<br>Accuracy of the instrument	
26		钝性/非钝性解剖性能<br>Blunt/non-blunt anatomical performance	
27		缝合性能<br>Stitch performance	
28		总体评价<br>Overall evaluation	

附录 5 远程手术网络传输记录表
Appendix 5 Remote Surgery Network Transmission Record

受试者筛选号：____________研究者签名：_____________签名日期：_______________
Subject screening number: Investigator's signature: Signature Date:

**1.术前网络测试情况**<br>1. Preoperative network test
网络带宽_______________Mbs<br>Network bandwidth Mbs
网络传输延时最大值_____________ms<br>Maximum value of network transmission delay ms<br>网络信号延时最小值_____________ms网络信号延时平均值___ms<br>Minimum network signal delay Average value of ms network signal delay ms
丢包率_____________%<br>Packet loss rate %
**2．术中网络切换及传输情况**<br>2. Network switching and transmission during operation
术中是否中途切换操作（含本地协作） □否 □是_________次切换原因：<br>Whether the operation is switched halfway during the operation (including local cooperation) No Yes Secondary switching reason:
术中是否中断网络 □否 □是_________次中断原因：<br>Whether the network is interrupted during the operation No Yes Reason for the second interruption:
术中是否出现网络原因导致系统故障或报警 □否 □是_________次故障或者报警原因：<br>Whether there is system failure or alarm caused by network during the operation No Yes Cause of fault or alarm:
网络传输延时最大值 ____________ms网络信号延时最小值___ms网络信号延时平均值_ms<br>Maximum value of network transmission delay Minimum value of ms network signal delay Average value of ms network signal delay ms<br>丢包率_____________% 定义：手术过程中，（丢包数量/传输总的包数量）*100%手术结束后，记录网络传输的延时相关数据。<br>Packet loss rate % Definition: During the operation, (number of packets lost/total number of packets transmitted) * 100% After the operation, record the delay related data of network transmission.

研究者声明
Investigator Statement

我同意：
I agre:

1.严格按照赫尔辛基宣言、中国现行法规、以及试验方案的要求进行本次临床试验。

The clinical trial was conducted in strict accordance with the requirements of the Declaration of Helsinki, current Chinese regulations, and the trial protocol.

2.将所要求的全部数据准确记录于病例报告表（CRF）中，配合完成临床试验报告。

Accurately record all the required data in the case report form (CRF) and cooperate with the completion of the clinical trial report.

3.试验医疗器械仅用于本次临床试验，在临床试验过程中完整准确地记录试验医疗器械的接收和使用情况，并保存记录。

The trial medical device is only used for this clinical trial. The receipt and use of the trial medical device shall be recorded completely and accurately during the clinical trial, and the records shall be kept.

4.允许申办者授权或派遣的监查员、稽查员和监管部门对该项临床试验进行监查、稽查和检查。

Allow monitors, inspectors and regulatory authorities authorized or dispatched by the sponsor to monitor, inspect and inspect the clinical trial.

5.严格履行各方签署的临床试验合同/协议条款。
Strictly implement the terms of the clinical trial contract/agreement signed by all parties.

我已全部阅读了临床试验方案，包括以上的声明，我同意以上全部内容。

I have read the entire clinical trial protocol, including the above statement, and I agree with all of the above.

主要研究者
Principal Investigator

由召
You Zhao

签名
Signature

必寸年/v月沭日
Bi Cun Nian/V Yue Shu Ri

医疗器械临床试验机构
Medical Device Clinical Trial Organization

签章
Signature and seal

年月日
Year, month and day

申办者
Sponsor

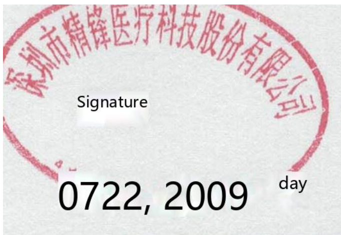

Supplement: Clinical Trial Protocol [file mmc1.pdf]
