# Supplementary material for: Feasibility and safety of remote robotic hepatectomy: a prospective single-arm study with MP1000 system in China
Source: eClinicalMedicine. 2025 Oct 22;89:103579. doi: 10.1016/j.eclinm.2025.103579 (PMC12589949; doi:10.1016/j.eclinm.2025.103579)
Supplement: Supplementary scale records [file mmc2.pdf]

NASA-TLX 量化表

受试者筛选号：02001 医生姓名：[illegible] 日期：2025.3.24

本研究采用 NASA-TLX（NASA_Task Load Index）量表对主刀医生操作腹腔内窥镜手术系统后的主观疲劳感和手术完成满意度进行评估，每一项评估指标划分为 21 个不同等级。

1. 脑力需求（完成任务过程中付出多少脑力活动？如思考、决定、计算、记忆、观察、搜查等。该工作从脑力方面对你而言是容易还是困难？简单还是复杂？要求严格还是不严格？）

非常低（1） 非常高（21）

2. 体力需求（完成任务过程中需付出多少体力？如：拖拽、旋转、控制、进行活动的程度等。该任务从体力方面对你而言是容易还是困难？是缓慢还是快速？肌肉感到松弛还是紧张？动作轻松还是费力？）

非常低（1） 非常高（21）

3. 时间需求（工作速率或节奏是缓慢并使人感到从容不迫，还是快速令人感到慌乱？）

非常低（1） 非常高（21）

4. 业绩水平（完成目标取得的成绩怎么样？对取得的成绩，您的满意程度有多大？）

非常低（1） 非常高（21）

5. 努力程度（你付出了多少努力来完成任务？）

非常低（1） 非常高（21）

6. 受挫程度（在执行任务时，你感到不安，沮丧、急躁、烦恼的程度有多大？）

非常低（1） 非常高（21）

远程手术操作评分表

受试者筛选号：02001 主刀医生：[illegible] 日期：2025.3.24

评分规则：5 分：非常满意，能够满足临床所有的需求。4 分：比较满意，能够满足临床基本的需求。3 分：基本满意，能够满足临床最低的需求。2 分：不太满意，不能满足临床最低的需求。1 分：很不满意，完全不能进行临床操作。

序号	一级指标	二级指标	评分
1	延迟稳定指标	内窥镜图像延迟	5
2		主从操作延迟	5
3		成像稳定性，无卡顿	5
4		视频交互延迟	5
5		语音交互延迟	3
6	交互质量评价	语音交互稳定性	3
7		语音交互清晰性	3
8		语音交互模式满意度	4
9		视频交互稳定性	5
10		视频交互清晰性	4
11		视频交互模式满意度	5
12	内窥镜图像质量	视野大小	5
13		清晰度	4
14		景深	5
15		分辨能力	5
16		立体感	5
17		镜头抗模糊的能力	4
18		抗反光的能力	5
19		图像/色彩保真性	5
20	手术操作	器械操作范围	5
21		器械灵活性	5
22		器械夹持	5
23		器械剪切力	5
24		器械电切	5
25		器械电凝	5
26		器械运动延迟性	5
27		器械精准度	5
28		钝性/非钝性解剖性能	5
29		缝合性能	5

NASA-TLX 量化表

受试者筛选号：02002 医生姓名：[illegible] 日期：2025.3.26

本研究采用 NASA-TLX（NASA_Task Load Index）量表对主刀医生操作腹腔内窥镜手术系统后的主观疲劳感和手术完成满意度进行评估，每一项评估指标划分为 21 个不同等级。

1. 脑力需求（完成任务过程中付出多少脑力活动？如思考、决定、计算、记忆、观察、搜查等。该工作从脑力方面对你而言是容易还是困难？简单还是复杂？要求严格还是不严格？）

非常低（1） 非常高（21）

2. 体力需求（完成任务过程中需付出多少体力？如：拖拽、旋转、控制、进行活动的程度等。该任务从体力方面对你而言是容易还是困难？是缓慢还是快速？肌肉感到松弛还是紧张？动作轻松还是费力？）

非常低（1） 非常高（21）

3. 时间需求（工作速率或节奏是缓慢并使人感到从容不迫，还是快速令人感到慌乱？）

非常低（1） 非常高（21）

4. 业绩水平（完成目标取得的成绩怎么样？对取得的成绩，您的满意程度有多大？）

非常低（1） 非常高（21）

5. 努力程度（你付出了多少努力来完成任务？）

非常低（1） 非常高（21）

6. 受挫程度（在执行任务时，你感到不安，沮丧、急躁、烦恼的程度有多大？）

非常低（1） 非常高（21）

远程手术操作评分表

受试者筛选号：02002 主刀医生：[illegible] 日期：2025.3.26

评分规则：5分：非常满意，能够满足临床所有的需求。4分：比较满意，能够满足临床基本的需求。3分：基本满意，能够满足临床最低的需求。2分：不太满意，不能满足临床最低的需求。1分：很不满意，完全不能进行临床操作。

序号	一级指标	二级指标	评分
1	延迟稳定指标	内窥镜图像延迟	5
2		主从操作延迟	5
3		成像稳定性，无卡顿	5
4		视频交互延迟	5
5		语音交互延迟	3
6	交互质量评价	语音交互稳定性	3
7		语音交互清晰性	3
8		语音交互模式满意度	3
9		视频交互稳定性	5
10		视频交互清晰性	4
11		视频交互模式满意度	4
12	内窥镜图像质量	视野大小	5
13		清晰度	4
14		景深	4
15		分辨能力	4
16		立体感	5
17		镜头抗模糊的能力	4
18		抗反光的能力	4
19		图像/色彩保真性	4
20	手术操作	器械操作范围	5
21		器械灵活性	5
22		器械夹持	5
23		器械剪切力	5
24		器械电切	5
25		器械电凝	5
26		器械运动延迟性	5
27		器械精准度	5
28		钝性/非钝性解剖性能	[illegible]

NASA-TLX 量化表

受试者筛选号：02003　　医生姓名：[illegible]　　日期：2025.3.28

本研究采用 NASA-TLX（NASA_Task Load Index）量表对主刀医生操作腹腔内窥镜手术系统后的主观疲劳感和手术完成满意度进行评估，每一项评估指标划分为 21 个不同等级。

1. 脑力需求（完成任务过程中付出多少脑力活动？如思考、决定、计算、记忆、观察、搜查等。该工作从脑力方面对你而言是容易还是困难？简单还是复杂？要求严格还是不严格？）

非常低（1）　　非常高（21）

2. 体力需求（完成任务过程中需付出多少体力？如：拖拽、旋转、控制、进行活动的程度等。该任务从体力方面对你而言是容易还是困难？是缓慢还是快速？肌肉感到松弛还是紧张？动作轻松还是费力？）

非常低（1）　　非常高（21）

3. 时间需求（工作速率或节奏是缓慢并使人感到从容不迫，还是快速令人感到慌乱？）

非常低（1）　　非常高（21）

4. 业绩水平（完成目标取得的成绩怎么样？对取得的成绩，您的满意程度有多大？）

非常低（1）　　非常高（21）

5. 努力程度（你付出了多少努力来完成任务？）

非常低（1）　　非常高（21）

6. 受挫程度（在执行任务时，你感到不安，沮丧、急躁、烦恼的程度有多大？）

非常低（1）　　非常高（21）

远程手术操作评分表

受试者筛选号：02003 主刀医生：[illegible] 日期：2025.3.28

评分规则：5 分：非常满意，能够满足临床所有的需求。4 分：比较满意，能够满足临床基本的需求。3 分：基本满意，能够满足临床最低的需求。2 分：不太满意，不能满足临床最低的需求。1 分：很不满意，完全不能进行临床操作。

序号	一级指标	二级指标	评分
1	延迟稳定指标	内窥镜图像延迟	5
2		主从操作延迟	5
3		成像稳定性，无卡顿	5
4		视频交互延迟	5
5		语音交互延迟	5
6	交互质量评价	语音交互稳定性	5
7		语音交互清晰性	5
8		语音交互模式满意度	5
9		视频交互稳定性	5
10		视频交互清晰性	3
11		视频交互模式满意度	4
12	内窥镜图像质量	视野大小	5
13		清晰度	3
14		景深	4
15		分辨能力	4
16		立体感	4
17		镜头抗模糊的能力	4
18		抗反光的能力	4
19		图像/色彩保真性	4
20	手术操作	器械操作范围	5
21		器械灵活性	5
22		器械夹持	5
23		器械剪切力	5
24		器械电切	5
25		器械电凝	5
26		器械运动延迟性	5
27		器械精准度	5
28		钝性/非钝性解剖性能	5
29		缝合性能	5

NASA-TLX 量化表

受试者筛选号：02004 医生姓名：[illegible] 日期：2025.4.2

本研究采用 NASA-TLX（NASA_Task Load Index）量表对主刀医生操作腹腔内窥镜手术系统后的主观疲劳感和手术完成满意度进行评估，每一项评估指标划分为 21 个不同等级。

1. 脑力需求（完成任务过程中付出多少脑力活动？如思考、决定、计算、记忆、观察、搜查等。该工作从脑力方面对你而言是容易还是困难？简单还是复杂？要求严格还是不严格？）

非常低（1） 非常高（21）

2. 体力需求（完成任务过程中需付出多少体力？如：拖拽、旋转、控制、进行活动的程度等。该任务从体力方面对你而言是容易还是困难？是缓慢还是快速？肌肉感到松弛还是紧张？动作轻松还是费力？）

非常低（1） 非常高（21）

3. 时间需求（工作速率或节奏是缓慢并使人感到从容不迫，还是快速令人感到慌乱？）

非常低（1） 非常高（21）

4. 业绩水平（完成目标取得的成绩怎么样？对取得的成绩，您的满意程度有多大？）

非常低（1） 非常高（21）

5. 努力程度（你付出了多少努力来完成任务？）

非常低（1） 非常高（21）

6. 受挫程度（在执行任务时，你感到不安，沮丧、急躁、烦恼的程度有多大？）

非常低（1） 非常高（21）

远程手术操作评分表

受试者筛选号：02004 主刀医生：[illegible] 日期：2025.4.2

评分规则：5 分：非常满意，能够满足临床所有的需求。4 分：比较满意，能够满足临床基本的需求。3 分：基本满意，能够满足临床最低的需求。2 分：不太满意，不能满足临床最低的需求。1 分：很不满意，完全不能进行临床操作。

序号	一级指标	二级指标	评分
1	延迟稳定指标	内窥镜图像延迟	5
2		主从操作延迟	5
3		成像稳定性，无卡顿	5
4		视频交互延迟	5
5		语音交互延迟	5
6	交互质量评价	语音交互稳定性	5
7		语音交互清晰性	5
8		语音交互模式满意度	5
9		视频交互稳定性	5
10		视频交互清晰性	3
11		视频交互模式满意度	4
12	内窥镜图像质量	视野大小	5
13		清晰度	3
14		景深	4
15		分辨能力	4
16		立体感	4
17		镜头抗模糊的能力	5
18		抗反光的能力	5
19		图像/色彩保真性	4
20	手术操作	器械操作范围	5
21		器械灵活性	5
22		器械夹持	4
23		器械剪切力	5
24		器械电切	5
25		器械电凝	5
26		器械运动延迟性	5
27		器械精准度	5
28		钝性/非钝性解剖性能	5
29		缝合性能	5

NASA-TLX 量化表

受试者筛选号：02005　　　　医生姓名：[illegible]　　　　日期：2025.4.16

本研究采用 NASA-TLX（NASA_Task Load Index）量表对主刀医生操作腹腔内窥镜手术系统后的主观疲劳感和手术完成满意度进行评估，每一项评估指标划分为 21 个不同等级。

1. 脑力需求（完成任务过程中付出多少脑力活动？如思考、决定、计算、记忆、观察、搜查等。该工作从脑力方面对你而言是容易还是困难？简单还是复杂？要求严格还是不严格？）

非常低（1）　　　　非常高（21）

2. 体力需求（完成任务过程中需付出多少体力？如：拖拽、旋转、控制、进行活动的程度等。该任务从体力方面对你而言是容易还是困难？是缓慢还是快速？肌肉感到松弛还是紧张？动作轻松还是费力？）

非常低（1）　　　　非常高（21）

3. 时间需求（工作速率或节奏是缓慢并使人感到从容不迫，还是快速令人感到慌乱？）

非常低（1）　　　　非常高（21）

4. 业绩水平（完成目标取得的成绩怎么样？对取得的成绩，您的满意程度有多大？）

非常低（1）　　　　非常高（21）

5. 努力程度（你付出了多少努力来完成任务？）

非常低（1）　　　　非常高（21）

6. 受挫程度（在执行任务时，你感到不安，沮丧、急躁、烦恼的程度有多大？）

非常低（1）　　　　非常高（21）

远程手术操作评分表

受试者筛选号：02005 主刀医生：[signature] 日期：2025.4.16

评分规则：5分：非常满意，能够满足临床所有的需求。4分：比较满意，能够满足临床基本的需求。3分：基本满意，能够满足临床最低的需求。2分：不太满意，不能满足临床最低的需求。1分：很不满意，完全不能进行临床操作。

序号	一级指标	二级指标	评分
1	延迟稳定指标	内窥镜图像延迟	5
2		主从操作延迟	5
3		成像稳定性，无卡顿	5
4		视频交互延迟	5
5		语音交互延迟	5
6	交互质量评价	语音交互稳定性	5
7		语音交互清晰性	5
8		语音交互模式满意度	5
9		视频交互稳定性	5
10		视频交互清晰性	4
11		视频交互模式满意度	5
12	内窥镜图像质量	视野大小	5
13		清晰度	4
14		景深	5
15		分辨能力	5
16		立体感	5
17		镜头抗模糊的能力	5
18		抗反光的能力	5
19		图像/色彩保真性	4
20	手术操作	器械操作范围	5
21		器械灵活性	5
22		器械夹持	5
23		器械剪切力	5
24		器械电切	5
25		器械电凝	5
26		器械运动延迟性	5
27		器械精准度	5
28		钝性/非钝性解剖性能	5
29		缝合性能	5

NASA-TLX 量化表

受试者筛选号：02006　　医生姓名：[illegible]　　日期：2025.4.18

本研究采用 NASA-TLX（NASA_Task Load Index）量表对主刀医生操作腹腔内窥镜手术系统后的主观疲劳感和手术完成满意度进行评估，每一项评估指标划分为 21 个不同等级。

1. 脑力需求（完成任务过程中付出多少脑力活动？如思考、决定、计算、记忆、观察、搜查等。该工作从脑力方面对你而言是容易还是困难？简单还是复杂？要求严格还是不严格？）

非常低（1）　　非常高（21）

2. 体力需求（完成任务过程中需付出多少体力？如：拖拽、旋转、控制、进行活动的程度等。该任务从体力方面对你而言是容易还是困难？是缓慢还是快速？肌肉感到松弛还是紧张？动作轻松还是费力？）

非常低（1）　　非常高（21）

3. 时间需求（工作速率或节奏是缓慢并使人感到从容不迫，还是快速令人感到慌乱？）

非常低（1）　　非常高（21）

4. 业绩水平（完成目标取得的成绩怎么样？对取得的成绩，您的满意程度有多大？）

非常低（1）　　非常高（21）

5. 努力程度（你付出了多少努力来完成任务？）

非常低（1）　　非常高（21）

6. 受挫程度（在执行任务时，你感到不安，沮丧、急躁、烦恼的程度有多大？）

非常低（1）　　非常高（21）

远程手术操作评分表

受试者筛选号：02006 主刀医生：[signature] 日期：2025.4.18

评分规则：5分：非常满意，能够满足临床所有的需求。4分：比较满意，能够满足临床基本的需求。3分：基本满意，能够满足临床最低的需求。2分：不太满意，不能满足临床最低的需求。1分：很不满意，完全不能进行临床操作。

序号	一级指标	二级指标	评分
1	延迟稳定指标	内窥镜图像延迟	5
2		主从操作延迟	5
3		成像稳定性，无卡顿	5
4		视频交互延迟	5
5		语音交互延迟	5
6	交互质量评价	语音交互稳定性	5
7		语音交互清晰性	5
8		语音交互模式满意度	5
9		视频交互稳定性	5
10		视频交互清晰性	4
11		视频交互模式满意度	5
12	内窥镜图像质量	视野大小	5
13		清晰度	4
14		景深	5
15		分辨能力	5
16		立体感	5
17		镜头抗模糊的能力	5
18		抗反光的能力	5
19		图像/色彩保真性	5
20	手术操作	器械操作范围	5
21		器械灵活性	5
22		器械夹持	5
23		器械剪切力	5
24		器械电切	5
25		器械电凝	5
26		器械运动延迟性	5
27		器械精准度	5
28		钝性/非钝性解剖性能	5
29		缝合性能	5
