# Supplementary material for: Feasibility and safety of remote robotic hepatectomy: a prospective single-arm study with MP1000 system in China
Source: eClinicalMedicine. 2025 Oct 22;89:103579. doi: 10.1016/j.eclinm.2025.103579 (PMC12589949; doi:10.1016/j.eclinm.2025.103579)
Supplement: Scoring table of telesurgery Chinese-English [file mmc3.docx]

远程手术操作评分表

版本号及日期：V01，2024 年10 月30 日

Scoring table of telesurgery Version and date: V01, Oct 30, 2024

**远程手术操作评分表**

Scoring table of telesurgery

受试者筛选号： 主刀医生： 日期：

Screening number: Surgeon: Date:

评分规则：5 分：非常满意，能够满足临床所有的需求。4 分：比较满意，能够满足

临床基本的需求。3 分：基本满意，能够满足临床最低的需求。2 分：不太满意，不能满足临床最低的需求。1 分：很不满意，完全不能进行临床操作。

Score rule: 1-5. Score 5: Very satisfied, meeting all clinical needs. Score 4: Quite satisfied, meeting most clinical needs. Score 3: Score 3: basically satisfied, meeting the minimum clinical needs. Score 2: Quite unsatisfied, unable to meet the minimum clinical needs. Score 1: Very unsatisfied, completely unable to operate.

| **序号**  **No.** | **一级指标**  **First grade index** | **二级指标**  **Second grade index** | **评分**  **Score** |
| --- | --- | --- | --- |
| 1 | 延迟稳定指标  Latency and stability  evaluation | 内窥镜图像延迟  Endoscopic image latency |  |
| 2 |  | 主从操作延迟  Master-slave operation latency |  |
| 3 |  | 成像稳定性，无卡顿  Imaging stability |  |
| 4 |  | 视频交互延迟  Video interaction latency |  |
| 5 |  | 语音交互延迟  Voice interaction latency |  |
| 6 | 交互质量评价  Interaction evaluation | 语音交互稳定性  Voice interaction stability |  |
| 7 |  | 语音交互清晰性  Speech interaction clarity |  |
| 8 |  | 语音交互模式满意度  Voice interaction mode |  |
| 9 |  | 视频交互稳定性  Video interaction stability |  |
| 10 |  | 视频交互清晰性  Video interaction clarity |  |
| 11 |  | 视频交互模式满意度  Video interaction mode |  |
| 12 | 内窥镜图像质量  Endoscopic image  evaluation | 视野大小  Field of view size |  |
| 13 |  | 清晰度  Definition |  |
| 14 |  | 景深  Depth of field |  |
| 15 |  | 分辨能力  Discriminative ability |  |
| 16 |  | 立体感  Three-dimensional sense |  |
| 17 |  | 镜头抗模糊的能力  Ability of the lens to resist blurring |  |
| 18 |  | 抗反光的能力  Anti reflective ability |  |
| 19 |  | 图像/色彩保真性  Image/Color fidelity |  |
| 20 | 手术操作  Operational stability  evaluation | 器械操作范围  Operating range of surgical instruments |  |
| 21 |  | 器械灵活性  Instrument flexibility |  |
| 22 |  | 器械夹持  Instrument clamping capability |  |
| 23 |  | 器械剪切力  Instrument shearing force |  |
| 24 |  | 器械电切  Electrocautery ablation ability |  |
| 25 |  | 器械电凝  Electrocautery coagulation ability |  |
| 26 |  | 器械运动延迟性  Instrumental motion latency |  |
| 27 |  | 器械精准度  Instrumental accuracy |  |
| 28 |  | 钝性/非钝性解剖性能  Blunt/non-blunt anatomical ability |  |
| 29 |  | 缝合性能  Suturing ability |  |
