# Supplementary material for: Feasibility and safety of remote robotic hepatectomy: a prospective single-arm study with MP1000 system in China
Source: eClinicalMedicine. 2025 Oct 22;89:103579. doi: 10.1016/j.eclinm.2025.103579 (PMC12589949; doi:10.1016/j.eclinm.2025.103579)
Supplement: Translated Abstract [file mmc4.docx]

The following translations in Chinese were submitted by the authors and we reproduce them as supplied. They have not been peer reviewed. Our editorial processes have only been applied to the original abstract in English, which should serve as reference for this manuscript.

**Summary**

**背景:**远程手术已经在某些场景中体现了许多优势。可是，考虑到肝切除术具有

高风险，目前仍然没有评价机器人远程肝切除术安全性和有效性的前瞻性研究。

本研究是世界上首个远程肝切除术的前瞻性单臂队列，用于评价应用MP1000远

程手术系统实施机器人辅助远程肝切除术的安全性和有效性。

**方法：**本研究由华中科技大学药物临床试验伦理委员会批准（批准号：[2024]324

号），并在中国临床试验登记网站登记（登记号：ChiCTR2500097679）。从

年3月1日至2025年4月18日连续招募了6名病人。经过了严格的术前评估

后，我们应用MP1000远程手术系统实施机器人肝切除术。本研究的首要目的是

评价手术成功率和并发症发生率。手术医生的术后任务负荷使用NASA-TLX量

表评价。

**发现：**远程系统的网络连接平滑。所有手术均成功完成，没有转变为开腹或本地

机器人辅助手术。手术医生报告了相当低的工作负荷。所有病人恢复良好并顺利

出院。可是，第二个病人在出院后30天内出现了菌血症并再次入院，使用抗生

素治疗康复后出院。

**解释：**机器人远程肝切除术是一种可行、有效的微创治疗方式。该方式可以为医

疗资源有限的地区提供高质量的医疗服务。然而，尚需进一步的多中心、大规模

的随机对照试验验证其有效性和安全性。

**基金：**本研究获得四大慢病国家科技重大专项课题（2023ZD0502001）、国家自然

科学基金面上项目（82473040）、湖北省技术攻关项目（2023BAA016-3）、同济

医院高质量临床研究基金（2024TJCR014）资助。
